# *Nxhl* Controls Angiogenesis by Targeting VE-PTP Through Interaction With Nucleolin

**DOI:** 10.3389/fcell.2021.728821

**Published:** 2021-10-11

**Authors:** Honglin Luo, Yongde Zhang, Yanfei Deng, Lequn Li, Zhaoan Sheng, Yanling Yu, Yong Lin, Xiaohan Chen, Pengfei Feng

**Affiliations:** ^1^Guangxi Key Laboratory for Aquatic Genetic Breeding and Healthy Aquaculture, Guangxi Academy of Fishery Sciences, Nanning, China; ^2^Department of Hepatobiliary Surgery, Affiliated Tumor Hospital of Guangxi Medical University, Nanning, China; ^3^College of Animal Science and Technology, Guangxi University, Nanning, China

**Keywords:** angiogenesis, *nxhl*, nucleolin, vascular, VE-PTP

## Abstract

Precise regulation of angiogenesis is required for organ development, wound repair, and tumor progression. Here, we identified a novel gene, *nxhl* (New XingHuo light), that is conserved in vertebrates and that plays a crucial role in vascular integrity and angiogenesis. Bioinformatic analysis uncovered its essential roles in development based on co-expression with several key developmental genes. Knockdown of *nxhl* in zebrafish causes global and pericardial edema, loss of blood circulation, and vascular defects characterized by both reduced vascularization in intersegmental vessels and decreased sprouting in the caudal vein plexus. The *nxhl* gene also affects human endothelial cell behavior *in vitro*. We found that *nxhl* functions in part by targeting VE-PTP through interaction with NCL (nucleolin). Loss of *ptprb* (a VE-PTP ortholo) in zebrafish resulted in defects similar to *nxhl* knockdown. Moreover, *nxhl* deficiency attenuates tumor invasion and proteins (including VE-PTP and NCL) associated with angiogenesis and EMT. These findings illustrate that *nxhl* can regulate angiogenesis via a novel *nxhl*–NCL–VE-PTP axis, providing a new therapeutic target for modulating vascular formation and function, especially for cancer treatment.

## Introduction

Angiogenesis, the process of new blood vessel formation, is orchestrated by various angiogenic factors. Angiogenesis plays critical roles in reproduction, organ development, and wound repair. Angiogenesis is closely related to various diseases, including a range of tumors and nonneoplastic diseases such as age-related macular degeneration (Handa et al., 2019), diabetic macular edema (Wells et al., 2015), rheumatoid arthritis (Smolen et al., 2020), and atherosclerosis (Souilhol et al., 2020). Judah Folkman has suggested that angiogenesis should be considered an “organizing principle” in biology (Folkman, 2007). This conceptualization shifts the focus of therapeutic strategies from tumor cell-centered to anti-angiogenesis centered (Grothey and Galanis, 2009). In the past several decades, anti-angiogenic therapeutics have focused on the vascular endothelial growth factor (VEGF)/VEGFR signaling axis. Various inhibitors of this axis have been approved for solid cancers by the US Food and Drug Administration (Wilke et al., 2014). Over 3000 anti-angiogenic drugs have been registered in clinical trials for the treatment of cancer and ocular neovascular diseases (Ferrara and Adamis, 2016). However, in the clinical setting, simply blocking the existing VEGF signaling pathway or other angiogenic pathways appears to be less effective for advanced cases and often causes treatment resistance (Giantonio et al., 2007). New drugs targeting novel angiogenic pathways are clinically necessary and highly desirable.

We are particularly interested in the vascular endothelial protein tyrosine phosphatase (VE-PTP, known as PTPRB in zebrafish), which is indispensable during mouse blood vessel development (Baumer et al., 2006; Dominguez et al., 2007; Hayashi et al., 2013) owing to overactivation of Tie2 and increased vessel enlargement (Baumer et al., 2006; Dominguez et al., 2007; Winderlich et al., 2009). Evidence suggests that VE-PTP plays a crucial role in angiogenesis, integrity of endothelial cell (EC) adherens junctions, and vascular homeostasis (Nawroth et al., 2002; Baumer et al., 2006; Nottebaum et al., 2008; Monaghan-Benson and Burridge, 2013; Vestweber, 2015). VE-PTP binds to Tie2 and negatively controls Tie2-induced vascular remodeling and angiogenesis by dephosphorylation (Winderlich et al., 2009). Suppressing VE-PTP, either by genetic deletion or specific VE-PTP inhibitors, results in activation of the ability of Tie2 to regulate angiogenesis (Shen et al., 2014; Souma et al., 2018). Nucleolin (NCL) is a highly conserved gene that is broadly expressed in ECs (Christian et al., 2003; Fogal et al., 2009) and is enhanced on the surface of activated angiogenic ECs and many different types of cancer cells. Plasma membrane NCL (Christian et al., 2003; Hubing et al., 2007) and cell surface NCL regulate angiogenesis and tumorigenesis via interactions with various ligands such as VEGF (Christian et al., 2003), epidermal growth factor receptor (EGFR) (Farin et al., 2011), endostatin (Koutsioumpa and Papadimitriou, 2014), and human epidermal growth factor receptor 2 (HER2; ErbB2 (Wolfson et al., 2016). Inhibition of cell surface NCL in ECs significantly suppresses EC migration and prevents capillary tubule formation (Huang et al., 2006). A variety of aptamers or antibodies targeting NCL are promising therapeutic agents and are under clinical investigation for cancer treatment (Romano et al., 2019). Both VE-PTP and NCL potentially function similarly in angiogenesis. Targeting the upstream genes that directly or indirectly interact with VE-PTP or NCL may be a promising strategy to overcome the limitations of current anti-VEGF agents.

We thus asked whether both VE-PTP and NCL are closely associated and how they are regulated in angiogenesis. We aimed to investigate this in a zebrafish model, as the zebrafish is an ideal model system for the study of vertebrate gene function, especially angiogenesis regulation, due to its vascular network formation and expression patterns of key genes being highly similar to those of humans and other vertebrates (Danninger and Gimona, 2000; Song et al., 2004). The virtually transparent embryos of this species, external development of the embryo, and the ability to produce various transgenic germ line fish as well as the ability to accelerate genetic studies by gene knockdown or overexpression make investigation of vertebrate gene function and vasculature manipulation in zebrafish feasible and cost-effective (Lawson and Weinstein, 2002). Its conserved angiogenic signaling pathways make the zebrafish an ideal system for angiogenesis research and anti-angiogenic or anti-cancer drug screening (Lyons et al., 1998; Tran et al., 2007). In addition, knockdown of gene function by MOs (Morpholino Oligonucleotides) is the most familiar genetic approach employed to study genes of interest in zebrafish (Sugano and Neuhauss, 2013). By interfering with the mRNA transcripts, MOs are not affected by the genetic compensation response that occurs with the genome editing techniques such as TALENs or CRISPR-Cas9 (Rossi et al., 2015; El-Brolosy et al., 2019). Previous research has shown that genetic compensation is induced by deleterious mutations but not by gene MO knockdown (Rossi et al., 2015).

Herein we identified a novel gene, *nxhl* (New XingHuo light), that is conserved in vertebrates and is co-expressed with keystone genes for embryonic development. We employed a zebrafish model and the MO knockdown technique to investigate the function of nxhl. We found that loss of nxhl in zebrafish caused global and pericardial edema, loss of blood circulation, and vascular defects. The results indicated that nxhl functions in part by targeting VE-PTP through interaction with nucleolin (NCL). Loss of ptprb (a VE-PTP ortholog) in zebrafish phenocopies nxhl deficiency. Moreover, nxhl deficiency attenuates tumor invasion and proteins associated with angiogenesis and epithelial-mesenchymal transition (EMT). These findings illustrate that nxhl can regulate angiogenesis via the novel nxhl–NCL–VE-PTP axis, and thus the results provide a new therapeutic target for modulation of angiogenesis, especially for cancer treatment.

## Materials and Methods

### RNAs Preparation and Sequencing (Golden Pompano Embryos)

For pooled transcriptome data, RNAs were extracted from the intestines, middle kidney, head kidney, muscle, heart, and eggs using an RNeasy Plus Mini kit (DP441, QIAGEN, China). The RNAs were pooled for library construction. For transcriptome analyses of embryo development, samples of the whole embryo at different development stages (from OSP to YAPS) were collected. The RNAs of each stage were also extracted using the RNeasy Plus Mini kit (DP441, QIAGEN, China). The development stages were accurately identified through continuous observation during fertilized egg development using a microscope. A photo of each stage was simultaneously taken during sample collection based on the shape of each embryo stage. The mRNA was enriched by Oligo (dT) and fragmented with fragmentation buffer. Only 200–700 bp fragments were selected for PCR amplification and sequencing in the fragment size selection step. The RNA-Seq libraries for all samples were prepared using a TruSeq Stranded mRNA Sample Prep Kit (RS-122-2103) (Illumina, United States) following the manufacturer’s instructions and then sequenced on the Illumina HiSeq^TM^ sequencing platform.

### Gene Expression Quantification

Reads with more than 20% low-quality bases (quality < 20) were filtered out after removing the adapter and primer sequences. The clean reads were mapped to the golden pompano reference genome by TopHat v2.0.13 (Trapnell et al., 2012). The gene expression levels were calculated by Cufflinks v2.2. (Trapnell et al., 2012) using default parameters. Gene expression levels were normalized by reads per million per kilobase (FPKM). The genes that had expression values greater than 0.1 FPKM were considered as expressed genes. The genes were regarded as differentially expressed genes (DGEs) if the fold change (FC) was greater than 2 or smaller than 0.5 and the FDR was < 0.05. Furthermore, the genes were examined for enrichment of KEGG and GO terms using Fisher’s exact test.

### Weighted Gene Co-expression Network Analysis

The WGCNA R package (Langfelder and Horvath, 2008) was used to analyze the co-expression gene network of the transcriptomic dataset of 19 embryonic stages with the parameters soft-power = 6 and minimum module size = 30. Genes expressed > 0.5 TPM in > 1% of the 57 samples (14,327 genes) were used as input. Genes with standard deviation/mean > 0.5 were removed, and a total of 10,998 genes were used to build the co-expression modules. The function hclust was used to produce a hierarchical clustering tree of genes with the parameter method = “average.” A soft power threshold of 6 was used to fit a scale-free topology. Adjacency and TOM (topological overlap measure) similarity matrices were generated based on the soft power threshold. Ten modules were identified through the cluster detection function cutreeDynamic in hierarchical clustering of the genes based on the TOM dissimilarity measure, and the minimum module size was set to 30. Function mergeCloseModules was used to merge highly correlated modules with similar expression profiles. Function signedKME was used to calculate the relationships between modules using the signed eigengene-based connectivity. Function corPvalueStudent was used to calculate the significance between the gene expression modules and trait via the Pearson method.

### Zebrafish Care and Maintenance

Adult wild-type AB strain zebrafish were maintained at 28.5°C with a 14 h light/10 h dark cycle (Westerfield, 1993). Five to six pairs of zebrafish were set up for natural mating. On average, 200–300 embryos were generated. Embryos were maintained at 28.5°C in fish water (0.2% Instant Ocean Salt in deionized water). The embryos were washed and staged according to published references (Kimmel et al., 1995). The establishment and characterization of the *(fli1a:EGFP)* transgenic lines have been described elsewhere (Nasevicius and Ekker, 2000; Lawson and Weinstein, 2002). The zebrafish facility at SMOC (Shanghai Model Organisms Center, Inc.) is accredited by the Association for Assessment and Accreditation of Laboratory Animal Care (AAALAC) International.

### Zebrafish Microinjections

Gene Tools, LLC^1^ designed the morpholino (MO). Antisense MOs (GeneTools) were microinjected into fertilized one-cell stage embryos according to standard protocols (Lawson and Weinstein, 2002). Translation-blocking (ATG-MO) and splice-blocking morpholinos of *nxhl* (zgc:113227, NM_001014319.2) and *ptprb* (NM_001316727.1), respectively, were designed. The standard control morpholino was used as a control MO (Gene Tools). The amounts of the MOs used for injection were as follows: control MO, ATG-MO, and splice-blocking-MO, 4 ng per embryo. The effectiveness of *nxhl* and *ptprb* knockdown was confirmed by quantitative real-time PCR (qPCR). The morpholinos and primers are listed in Supplementary Table 1.

### Zebrafish Angiogenesis Studies and Image Acquisition

To evaluate angiogenesis development in zebrafish, fertilized one-cell *fli1a-EGFP* transgenic line embryos were injected with 4 ng *nxhl*-e1i1-MO, *nxhl*-ATG-MO, or control-MO, and *ptprb*-e4i4-MO, *ptprb*-ATG-MO, or control-MO. At 2 dpf (days post fertilization) the embryos were dechorionated and anesthetized with 0.016% MS-222 (tricaine methane sulfonate, Sigma-Aldrich, St. Louis, MO). Zebrafish were then oriented on the lateral side (anterior, left; posterior, right; dorsal, top), and mounted with 3% methylcellulose in a depression slide for observation by fluorescence microscopy. The phenotypes of complete intersegmental vessels (ISVs), parachordal vessels (PAV), and caudal vein plexus (CVP) were analyzed. Embryos and larvae were analyzed with a Nikon SMZ 1500 Fluorescence microscope and subsequently photographed with digital cameras. A subset of images was adjusted for levels of brightness, contrast, hue, and saturation with Adobe Photoshop 7.0 software (Adobe, San Jose, California) to optimize the visualization of the expression patterns. Quantitative image analyses were performed using image-based morphometric analysis (NIS-Elements D3.1, Japan) and ImageJ software (U.S. National Institutes of Health, Bethesda, MD, United States).^2^ Inverted fluorescent images were used for processing. Positive signals were defined by particle number using ImageJ. Ten animals for each treatment were quantified, and the total signal per animal was averaged.

### Quantitative Real-Time PCR

Total RNA was extracted from 30 to 50 embryos per group in Trizol reagent (Cat. No. 11667157001, Roche) according to the manufacturer’s instructions. RNA was reverse transcribed using the PrimeScript RT Reagent Kit with gDNA Eraser (Cat. No. RR047A, Takara). Quantification of gene expression was performed in triplicate using Bio-rad iQ SYBR Green Supermix (Cat. No. 1708880, Bio-rad) with detection on the Realplex system (Eppendorf). Relative gene expression quantification was based on the comparative threshold cycle method (2^–ΔΔCt^) using *ef1*α or β-actin as an endogenous control gene. qPCR on HUVECs was performed using similar procedures. All of the primers are listed in Supplementary Table 1.

### RNA-Seq (Zebrafish Embryos)

Control MO-injected embryos and embryos injected with *nxhl* MO at 3 dpf were frozen for RNA-seq analysis. Three biological replicates of 30 embryos each were analyzed in each group. RNA was purified using an RNAqueous Total RNA isolation kit (Cat. No. AM1912, Thermo Fisher Scientific). Libraries were prepared with a TruSeq RNA Library Prep kit v2 (Cat. No. RS-122-2001, Illumina) according to the manufacturer’s protocol. Libraries were sequenced at the CCHMC Core Facility using an Illumina HiSeq 2500 device (Illumina) to generate 75 bp paired-end reads. The quality of the RNA-Seq reads was checked using Fastqc.^3^ All of the low-quality reads were trimmed using trimmomatic.^4^ The trimmed RNA-Seq reads were mapped and quantified to the latest zebrafish genome assembly GRCz10 for each sample at default thresholds using RSEM.^5^ The mRNA levels were identified using TopHat v2.0.9 and Cufflinks and normalized by the Fragments Per Kilobase of exon model per Million mapped reads (FPKM). Differential expression was analyzed by using CSBB.^6^ Criteria of false discovery rate (FDR) < 0.01 and fold change < 0.5 or > 2.0 (<−1 or > 1 log_2_ ratio value, *p*-value < 0.05) were used to identify differentially expressed genes. Gene Ontology (GO) annotation, domain annotation, and Kyoto Encyclopedia of Genes and Genomes (KEGG) pathway annotation and enrichment were performed using ToppGene.^7^

### Transwell Migration and Invasion Assays

To examine the function of *nxhl* (the homolog of Harbi1), siRNA targeting human Harbi1 gene (NM_173811.4) was designed and synthesized (see Supplementary Table 1). HUVEC cells (ATCC, Manassas, Virginia, United States) were cultured in DMEM/F12 (Hyclone, United States) with 10% FBS (Gibco BRL. Co., Ltd.) and 1% penicillin-streptomycin (Sangon Biotech, China.) at 37°C in a 5% CO_2_ incubator. Three experimental groups, untreated controls, Ctrl-siRNA, and *nxhl*-siRNA were set up, and 30 pmol *nxhl*-siRNA per well in the 24-well plates (Corning-costa, United States) was transferred using 9 μl Lipofectamine RNAi MAX Reagent (Cat. No. 13778-075, Invitrogen, United States). The cell migration and invasion capacity of *nxhl* in HUVECs cells were determined by transwell insert chambers (Corning, NY, United States) covered with or without 50 μl of Matrigel (1:3 dilution, BD, NJ, United States). Cells were then harvested and dissociated into a single-cell suspension. Then, 5 × 10^4^ cells in serum-free medium were added to the upper chamber, and 600 μl of 20% FBS-containing medium was added to the lower chamber. The chambers were then incubated for 72 h (5% CO_2_, 37°C). Cells in the upper chamber were discarded, while cells in the lower chamber were fixed with 4% paraformaldehyde for 30 min and then stained with 0.1% crystal violet for 10 min. Cells that underwent migration or invasion were counted in triplicate in microscopic fields. The migration of nucleolin (NCL,NM_005381.3) was also examined by a similar protocol. All cancer cell lines used in this study, including HepG2 (hepatocellular carcinoma, HCC), A549 (non-small cell lung cancer, NSCLC), and HCT116 (colon carcinoma, Colo) were kept in our labs and identified by the short tandem repeat (STR) method. The siRNA for NCL is listed in Supplementary Table 8.

### Cell Proliferation Assay

The MTT assay was used to determine the proliferation of HCC cells. HCC cells were grown in DMEM with 10% FBS. Cells were counted using Trypan blue and a hemocytometer. Approximately 1 × 10^5^ HCC cells/well were seeded into 96-well plates. Three groups were set up in triplicate, an *nxhl*-siRNA group, a Ctrl-siRNA group, and an untreated control group. All cells were incubated for 0, 3, 6, 12, 18, 24, 36, and 48 h after being seeded with 50 μL MTT (Sigma Chemicals, MO) at 37°C in a 5% CO_2_ incubator. After incubation, all cells were treated with MTT solvent for 20 min at room temperate. The supernatants were removed from the cells, and DMSO was added. The cell proliferation was recorded by measuring the optical density (OD) at 570 nm.

### Colony Formation Assay

For this assay, 1 × 10^6^cells/well were planted and cultured in 6-well plates. *nxhl*-siRNA, Ctrl-siRNA and untreated control groups were set up in triplicate. The cells were cultured for 2 weeks, and the culture medium was refreshed every 3 days. The colonies were fixed with 4% polyoxymethylene for 15 min and stained with 0.1% crystal violet solution for 15 min. Colonies in each well were counted independently and photographed under a microscope.

### Tube Formation Assay

The HUVECs’ culture conditions and experimental setup were identical to those of the transwell migration and invasion assays. Thirty picomoles of *nxhl*-siRNA or NCL-siRNA per well of 24-well plates (Corning Costa, United States) were transferred by using 9 μl Lipofectamine RNAi MAX Reagent (Cat. No. 13778-075, Invitrogen, United States). Matrigel (250 μl per well, BD Biosciences, United States) was added to the plates and cultured at 37°C for 30 min. Then, a suspension containing 5 × 10^4^ HUVECs was added to each well and cultured at 37°C in a 5% CO_2_ incubator. A final concentration of 50 μM Calcein-AM (Cat. No. C8950, Solarbio, China) per well was added to the plates and incubated for 30 min at 37°C. Six hours later, the tube formation was observed under a fluorescence microscope. The number of formed tubes represented the tube forming capability of HUVECs.

### Comprehensive Identification of RNA-Binding Proteins by Mass Spectrometry

Zebrafish embryos (3 dpf) were collected, and a total of 2 × 10^7^ cells were prepared and re-suspended in precooled PBS buffer followed by crosslinking with 3% formaldehyde for 30 min at 25°C. The reaction was stopped by incubation with 0.125 M glycine for 5 min. After centrifugation at 1,000 RCF for 3 min, the pre-binding probes (100 pmol per 2 × 10^7^ cells; for probes see Supplementary Table 1) were incubated with streptavidin beads for 30 min. The unbound probes were removed by washing three times. The beads with probes were incubated with the cell lysate and hybridized at 37°C with shaking overnight. All of the beads were washed three times with pre-warmed wash buffer for 5 min. A small aliquot (1/20 of the beads) of post-ChIRP beads was reserved for RNA extraction and qPCR analysis. Then, 100 μL of elution buffer (12.5 mM biotin, 7.5 mM HEPES pH 7.5, 75 mM NaCl, 1.5 mM EDTA, 0.15% SDS, 0.075% sarkosyl, 0.02% Na-Deoxycholate, and 20 U benzonase) was added, and the protein was eluted at 37°C for 1 h. The eluent was transferred to a fresh low-binding tube, and the beads were eluted again with 100 μL of elution buffer. The two eluents were combined, and reverse-crosslinking was performed at 95°C for 30 min. The proteins were precipitated with 0.1% SDC and 10% TCA by centrifugation at 4°C for 2 h. The pellets were then washed with precooled 80% acetone three times before the proteins were used for mass spectrometry (MS) analysis. Then, 5 μL peptides of each sample were collected and separated by a nano-UPLC liquid phase system (easy-nLC1200) before they were detected using an on-line mass spectrometer (Q-Exactive) at a resolution of 70,000. All of the original MS data were queried against the zebrafish protein database (UNIPROT_zebrafish_2016_09). Only those proteins with an FDR < 0.01 and an adjusted *p*-value < 0.05 were considered differentially expressed. The identified proteins were then further examined using bioinformatics, including GO and KEGG pathway annotations.

### *Nxhl* Protein Expression and Antibody Preparation

Briefly, the *nxhl* gene (zgc:113227, NM_001014319.2) was synthesized, and the expression plasmid pET-B2m-*nxhl*-His was constructed using seamless cloning technology. The plasmid was transferred into the *Escherichia coli* strain B21 (DH3) for protein expression, and the resulting protein was purified using an Ni-NTA chromatography column. The purified *nxhl* protein was used to immunize Japanese big-eared rabbits to produce the polyclonal antibody. The specificity of the polyclonal antibody was detected by anti-His Western blotting, and its immune action was verified by ELISA. We purified 6 mg of fusion protein (62.0 kDa) with 85% purity. After immunization in rabbits, an *nxhl* polyclonal antibody with a titer of 1:256,000 was obtained. The concentration of the *nxhl* antibody purified by Protein G affinity chromatography column was 10 mg/mL, and the purity was 90%. The obtained *nxhl* antibody was used to perform Western blotting assays.

### Western Blotting Assays

Zebrafish tissues from the knockdown and control groups were treated with 1 mL of tissue lysate (50 mmol/L Tris, 0.1% SDS,150 mmol/L NaCl, 1% NP-40, 5 mmol/L EDTA, 5 μg/mL aprotinin and 2 mmoL/L PMSF followed by lysis with protein lysate at 4°C for 30 min). All of the samples were centrifuged at 12,000 r/min at 4°C for 20 min, and the supernatant was removed to detect the protein concentration using a bicinchoninic acid (BCA) kit (CWBIO. Co., Ltd., Shanghai, China). Samples were resolved by SDS-PAGE using a NuPAGE 4–12% gel (Life Technologies). Proteins were transferred onto a nitrocellulose filter (BioRad, Hercules, CA, United States) and sealed at 4°C overnight with 5% dried skim milk. The membranes were incubated with diluted primary rabbit polyclonal *ptprb* (PA5-68309, Invitrogen, United States) (1:1,000), Nucleolin (ab50279, Abcam, United Kingdom) (1:1,000), Nucleolin phosphor T76 (ab168363, Abcam, United Kingdom) (1:1000), *nxhl* (Lab made, 1:1000) and *ptprb* (Lab made, 1:1,000), Slug (ab27568, Abcam, United Kingdom) (1:500), N-cadherin (ab245117, Abcam, United Kingdom) (1:1,000), E-cadherin (20874-1-AP, Proteintech, China) (1:5,000), vimentin (10366-1-AP, Proteintech, China) (1:2,000), GAPDH (ab181602, Abcam, United Kingdom) (1:1,000) and β-actin (ab8226, Abcam, United Kingdom) (1:1,000) antibodies separately overnight at 4°C followed by washing with PBS at room temperature. The membranes were treated with goat-anti-rabbit, rabbit-anti-goat, or goat-anti-mouse IgG-HRP secondary antibody (1: 2,000, CWBiotech., Ltd., Beijing, China) and incubated at 37°C for 2 h. After washing with PBS, the membrane was soaked in enhanced chemiluminescence (ECL) reagent (CW Biotech., Ltd., Beijing, China) to visualize the signals of protein bands according to the manufacturer’s protocols. The β-actin protein levels were used as an internal control.

### RNA Binding Protein Immunoprecipitation Assay (RIP)

To examine the interactions of the nucleolin protein with *nxhl* mRNA and VE-PTP mRNA, RIP was conducted as follows: the constructed *nxhl*-pcDNA3.1 (pcDNA3.1 vector V79020, Invitrogen, United States) was transferred into 293T cells, and its overexpression was verified by qPCR. Then, 1 × 10^7^ 293T cells were suspended and lysed for 20 min with 1 ml RIPA lysis buffer (Thermo Fisher Scientific, United States) containing 1 μl of protease inhibitor (Beyotime, China). Twenty microliters of cell lysates were used as input. Magnetic beads were pretreated for RIP with an anti-rabbit IgG (Beyotime, China; negative control) isotype control and anti-nucleolin (ab50279, Abcam, United Kingdom) for 1 h at room temperature, and cell extracts were immunoprecipitated with the beads-antibody complexes at 4°C overnight. The retrieved RNA was isolated and purified by using the phenol-chloroform method and subjected to real-time qPCR and general reverse-transcription PCR for *nxhl* and VE-PTP analyses. The primers are listed in Supplementary Table 1.

### RNA Pull-Down Assay

To detect the interactions between VE-PTP mRNA and nucleolin protein in 293T cells and between *ptprb* mRNA and nucleolin protein in zebrafish tissues, probes for VE-PTP (human) and *ptprb* (zebrafish) were designed and synthesized. The probes are listed in Supplementary Table 1. Probes were labeled with 3 μg biotin and then heated at 95°C for 2 min followed by standing at room temperature for 30 min. Magnetic beads were washed and resuspended in 50 μl RIP buffer, and the biotinylated and denatured probes were added and incubated for 1 h at room temperature. The nucleolin protein was extracted with 1 ml RIP buffer, sonicated, centrifuged at 12,000 rpm for 15 min, and the supernatant (nucleolin protein) was retained. A magnetic separator was used to remove the liquid, and the protein solution was rinsed three times using RIP buffer. The protein solution was added to the magnetic bead-probe mixture, and RNase inhibitor was added to the lysate. The mixture was incubated at room temperature for 1 h and washed five times with 1 ml RIP buffer. Then, 2 × SDS loading buffer was added to the mixture, denatured at 95°C for 10 min, and used for subsequent Western blotting. The Western blotting was performed as described above. The antibody Nucleolin (ab50279, Abcam, United Kingdom) (1:1,000) was used in the detection of VE-PTP (human) and *ptprb* (zebrafish) in 293T cells and zebrafish tissue.

### Statistical Analysis

All data are presented as mean ± SEM. Statistical analysis and graphical representation of the data were performed using GraphPad Prism 7.0 (GraphPad Software, San Diego, CA). Statistical evaluation was performed by using Student’s *t*-tests, ANOVA, or χ^2^ tests as appropriate. A *p-*value of less than 0.05 was considered statistically significant. Statistical significance is indicated by ^∗^ or by *p-*value. An asterisk (^∗^) represents *p* < 0.05; ^∗∗^ indicates *p* < 0.001, and ^∗∗∗^ indicates *p* < 0.0001. The results are representative of at least three independent experiments.

## Results

### Evolutionary Conservation of *Nxhl* in Vertebrates

**FIGURE 1:**
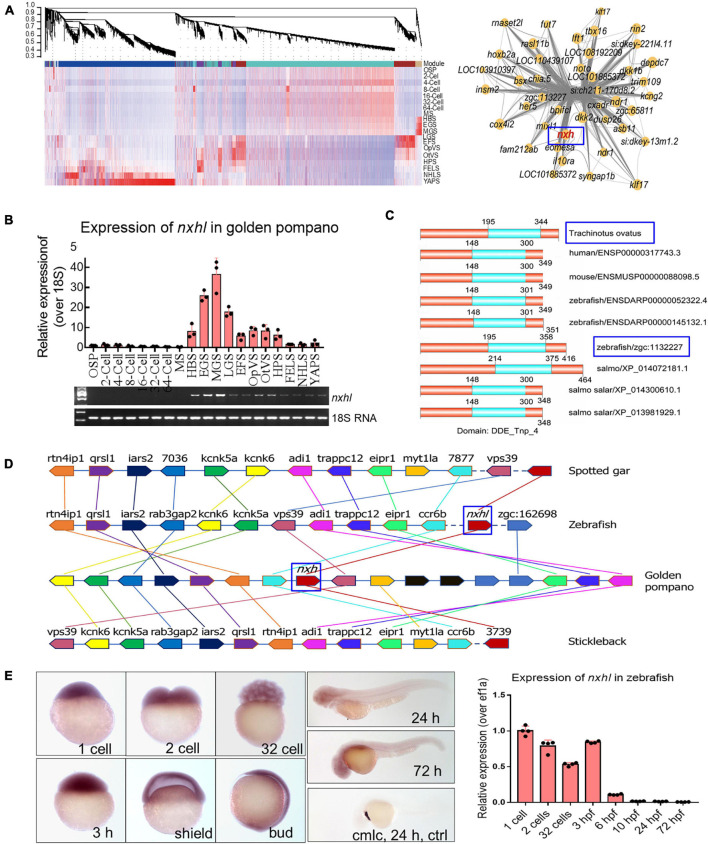
Evolutionary conservation of *nxhl* in vertebrate. **(A)** WGCNA analysis of embryonic development stages revealed gene-network modules enriched. HBS, EGS and MGS are classified into one group. OSP, oosperm; MS, morula stage; HBS, high blastula stage; EGS, early gastrula stage; MGS, middle gastrula stage; LGS, late gastrula stage; EFS, embryo formed stage; OpVS, optic vesicle stage; OtVS, otocyst vesicle stage; HPS, heart pulsation stage; FELS, formation of eye lens; NHLS, newly hatched larvae; YAPS, yolk absorption period. **(B)** Validation of expression level for *nxhl* by qPCR technology. 18s RNA was considered as internal marker. Gene structure of *nxhl* was showed at upper region. **(C)** Domains of *nxhl* and other homologous protein. The domain DDE_Tnp_4 was identified in SMART database (http://smart.embl.de/). **(D)** Micro-synteny analysis of *nxh* locus among spotted gar, zebrafish, gold pompano and stickleback. Two inversions and one insertion occurred in *nxh* locus region of golden pompano genomes. **(E)** The distribution of *nxhl* in zebrafish embryo by using whole-mount embryo *in situ* hybridization (WISH) and qPCR. Cmlc was used as positive control. *Nxhl* is mainly distributed in maternal developmental stages in zebrafish embryo.

We analyzed the gene expression pattern in teleost golden pompano embryos based on the transcriptome data (Supplementary Figure 1 and Supplementary Tables 2–7) and found that all 57 samples covering 19 developmental stages were separated into two components (Supplementary Figure 1). The first 33 samples (from OSP to MGS) clustered into a clade, and the residual 24 samples (from LGS to YAPS) clustered into another clade. The genes in the first clade were non-redundant reserved hub genes and were clearly “silenced” compared with those in the second clade in which the gene levels showed an explosive increase. We also noticed that before LGS, a group of genes in three stages, HBS, EGS, and MGS, were highly expressed in the first clade (Supplementary Figure 1). We clustered these genes using the WGCNA R package (The R Project for Statistical Computing, Vienna, Austria) and found that most of these clustered into the purple_module and were co-expressed in a close network, indicating regulatory roles of these genes (Figure 1A). EVM0008813 (designated New XingHuo light, *nxhl*; Figure 1B) was dominantly expressed in the HBS, EGS, and MGS stages; it was closely co-expressed with several key genes (Figure 1A) such as eomesa, dkk2, and mixl1 that play essential roles in embryonic development (Hart et al., 2002; Du et al., 2012; McGraw et al., 2014). We therefore propose that *nxhl* could be a crucial controller that regulates key steps in embryonic development. We found that *nxhl* contained two exons with two introns, and its expression (qPCR) in EGS, MGS, and LGS was highly matched to our sequencing data (FPKM, Figure 1B). We aligned the *nxhl* protein sequence with human, mouse, zebrafish, and salmon and found that this gene is conserved in vertebrate evolution (Figure 1C). We also found that only one gene, *zgc:113227* (zebrafish *nxhl*), shared 54.7% similarity to *nxhl* at the amino acid level in zebrafish. Both *nxhl* and zebrafish *nxhl* have the same functional domain DDE_Tnp_4 as the other seven genes in different species (Figure 1C), suggesting that they are conserved and may have similar biological functions. We subsequently confirmed that *nxhl* is the only homolog gene in zebrafish by collinear analysis, indicating that the function of *nxhl* could be investigated by using its homolog in a zebrafish model (Figure 1D). We then checked the distribution of *nxhl* in zebrafish embryos by the whole-mount embryo *in situ* hybridization (WISH) method, and the results showed that *nxhl* was mainly distributed in 1-cell, 2-cell, 32-cell, and 3 hpf stages; very little was detected in other stages, suggesting that it is a typical maternal gene (Figure 1E).

### *Nxhl* Affects Angiogenic Phenotypes *in vivo*

**FIGURE 2:**
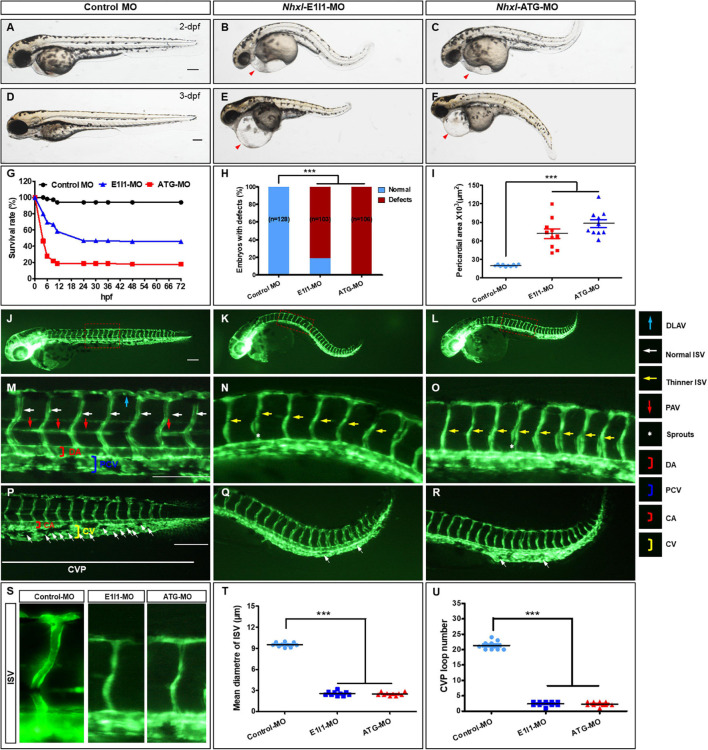
*Nxhl* regulates vascular development *in vivo***. (A–F)** Gross morphology at 2-dpf and 3-dpf. Compared with control zebrafish, *nxhl* knock-down causes pericardial oedema (**B,C,E,F**, red arrowheads) and circulation defects. Heart beat and circulation in caudal vein (CV) is visible in the control fish, but is abnormal in *nxhl* morphants (Supplementary Videos 1, 2). **(G)** A time-course plot of percent survival in control vs. *nxhl* morphants for 3 days. **(H)** Shows the percentage of embryos with development defects. **(I)** Quantification of the pericardial area of embryos. Error bars, mean ± SEM; ***p < 0.0001 (*n* = 10; ANOVA). **(J–R)** Representative fluorescent images of *Tg(fli1a:EGFP)^y1^* embryos at 2-dpf. **(J,M)** Image of trunk regions taken at 2-dpf, with the vascular structures visualized by eGFP fluorescence and labeled ISV and DLAV showed regular development in the embryo injected with control MO. The boxed regions are shown at higher magnification in the bottom panels. **(K,L,N,O)** Compared with control MO, embryos injected with *nxhl*-MO present a lower number of incomplete ISVs and thinner ISVs (**N,O**, yellow arrows) and ectopic sprouts (asterisk) of dorsal aorta. In control embryos, the parachordal vessels (PAV) form normally (**M**, red arrows). Compared with control, MO knock down *nxhl* prevents the parachordal vessels (PAV) formation, the precursor to the lymphatic system. In control embryos, caudal vein plexus (CVP) were formed honeycomb-like structures at the tail around 2-dpf (P, white arrows). In contrast, *nxhl* knock down resulted in specific defects in caudal vein plexus (CVP) formation **(Q,R)**. **(S–U)** Quantification of the mean diameter ISVs **(S,T)** and loop formation at CVP (U) shows significantly decrease in *nxhl* morphants. Columns, mean; SEM (*n* = 10; ANOVA) ***p < 0.0001. DLAV, dorsal longitudinal anastomotic vessels; ISV, intersegmental vessel; DA, dorsal aorta; PCV, posterior cardinal vein; CVP, caudal vein plexus; CA, caudal artery; CV, caudal vein; dpf, days post fertilization. Scale bar, 100 μm.

We next investigated the roles of *nxhl*. Firstly, we investigated whether loss of *nxhl* affected morphological development in zebrafish. The zebrafish *nxhl* gene was targeted in a specific morpholino antisense strategy to prevent the splicing of exon 1 (E1I1-MO). ATG-MO was used as a positive control. As designed, cleanly skipping exon 1 should frameshift the downstream sequence, leading to a premature termination codon in-frame that may trigger nonsense-mediated decay of the *nxhl* transcript (Supplementary Figure 2). We found that both the *nxhl* ^e1i1^ and *nxhl*^ATG^ morphants resulted in nearly identical phenotypes of pericardial edema, body axis bending, and circulation and caudal fin defects (Figures 2A–F and Supplementary Figures 2, 3 and Supplementary Videos 1, 2) with significantly lower survival rates (45.78%, *n* = 225 embryos in *nxhl* ^e1i1^ MO; 17.68%, *n* = 198 embryos in *nxhl*^ATG^ MO) compared with controls (*n* = 218 embryos) (Figures 2G,H). The pericardial area of embryos at 3 days post fertilization compared with control fish is shown in Figure 2I. Both nxhl e1i1 and nxhl ATG morphants dramatically disrupted normal splicing of *nxhl* (Figures 2A–F), indicating high efficiency and specificity of the morpholino knockdown of nxhl together with the fact that the gel band in *nxhl*-MO was weaker than in the control MO (Supplementary Figure 2), confirming that the phenotype of *nxhl* knockdown was *nxhl*-specific rather than an off-target effect (Figures 2A–F). Consistent with this, nxhl morphants resulted in a high percentage of embryos with defects (81.55%, n = 103 embryos in *nxhl* e1i1 MO and 100%, *n* = 106 embryos in *nxhl* ATG MO) at 3-dpf (Figure 2H and Supplementary Figure 2). This confirmed that loss of *nxhl* caused the pericardial edema and circulation and caudal fin defects in the zebrafish. We then used the transgenic fluorescent Tg(fli1a:EGFP)y1 zebrafish as a model to investigate connections between the vascular system and these phenotypes. Compared with control MO, embryos injected with *nxhl*-MO presented a low number of incomplete ISVs and thinner ISVs (Figures 2N,O, yellow arrows) and ectopic sprouts (asterisk) of the dorsal aorta, and knockdown of *nxhl* prevented the formation of the parachordal vessels (PAV) compared with control embryos. Moreover, in control embryos the caudal vein plexus (CVP) formed honeycomb-like structures at the tail around 2 dpf (Figure 2P, white arrows), while *nxhl* knockdown resulted in specific defects in CVP formation (Figures 2J–S). Quantification of the mean diameter of ISVs and loop formation at the CVP showed significant decreases in *nxhl* e1i1 morphants (*n* = 10 embryos) at 2 dpf (Figures 2T,U). Our data indicate that nxhl plays a critical role in controlling PAV, ISV, and CVP formation and vascular integrity during angiogenesis; this is highly consistent with the observed heart and caudal fin phenotypes.

### *Nxhl* Affects Angiogenic Phenotypes *in vitro*

**FIGURE 3:**
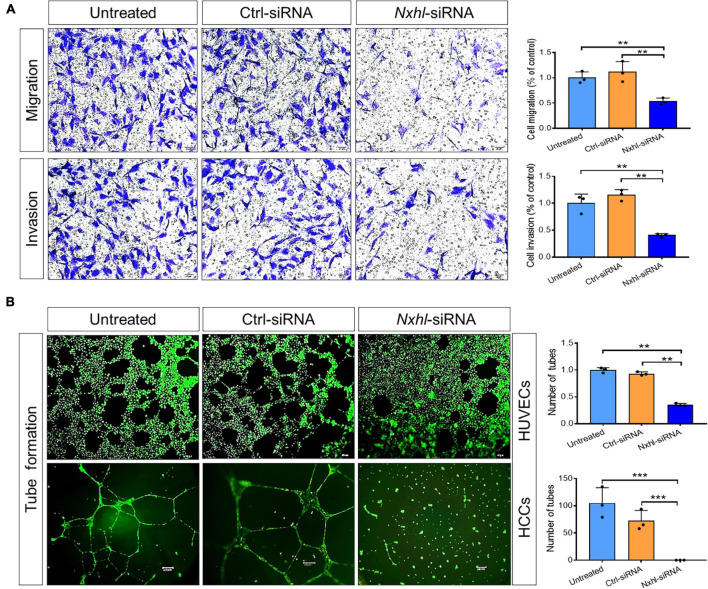
*Nxhl* affects angiogenic phenotypes *in vitro*. **(A)** Silence of *nxhl* (homolog of Harbi1) gene suppresses HUVEC cell migration and invasion. Representative images of migrated and invasive cells stained with crystal violet and inhibition of migration in *nxhl*-siRNA treated HUVEC cells. The migration and invasive potential of HUVECs treated with *nxhl*-siRNA was determined using transwell chambers as described in the “Materials and methods” section. **(B)** Silence of *nxhl* gene suppresses HUVEC and HCC cell tube formation abilities. Representative images of tube formation in *nxhl*-siRNA treated HUVEC cells and inhibition of tube formation *in vitro* (up row). Images were taken 6 h after addition of the supernatant. Tube networks were quantified using the Image J software and expressed as branches. Scale bars, 50 μm (middle row). Representative images of tube formation in *nxhl*-siRNA treated HCC cells (down row). Scale bars, 100 μm. Images were taken 24 h after addition of the supernatant. Tube networks were quantified using the Image J software and expressed as branches. The data represent as mean ± SEM from three independent experiments. *p < 0.05, **p < 0.001, and ***p < 0.0001 represents statistically significant.

Next, we examined the angiogenic functions of *nxhl in vitro* to find out whether the human homolog of *nxhl* plays a role in angiogenesis. As ECs line the inner lumen of vessels and are the elements upon which blood vessels are formed, we speculated that *nxhl* may affect angiogenesis via ECs. This was supported by significant enrichment of the genes involved in blood vessel morphogenesis upon *nxhl* depletion (Supplementary Table 2). To examine whether *nxhl* may function via ECs, we next silenced *nxhl* in HUVECs using a small interfering RNA (siRNA) targeting *nxhl*. Silence of *nxhl* (about 70% knockdown efficiency, Supplementary Figure 4) significantly inhibited tube formation and cell migration in comparison with controls. Furthermore, the invasion abilities in *nxhl–*defective cells were significantly inhibited compared with controls (Figure 3). This highlights the anti-angiogenesis function of *nxhl*. We considered that *nxhl* likely mediates angiogenic behavior via ECs.

### *Nxhl* Regulates *Ptprb* Expression and Angiogenic Networks

**FIGURE 4:**
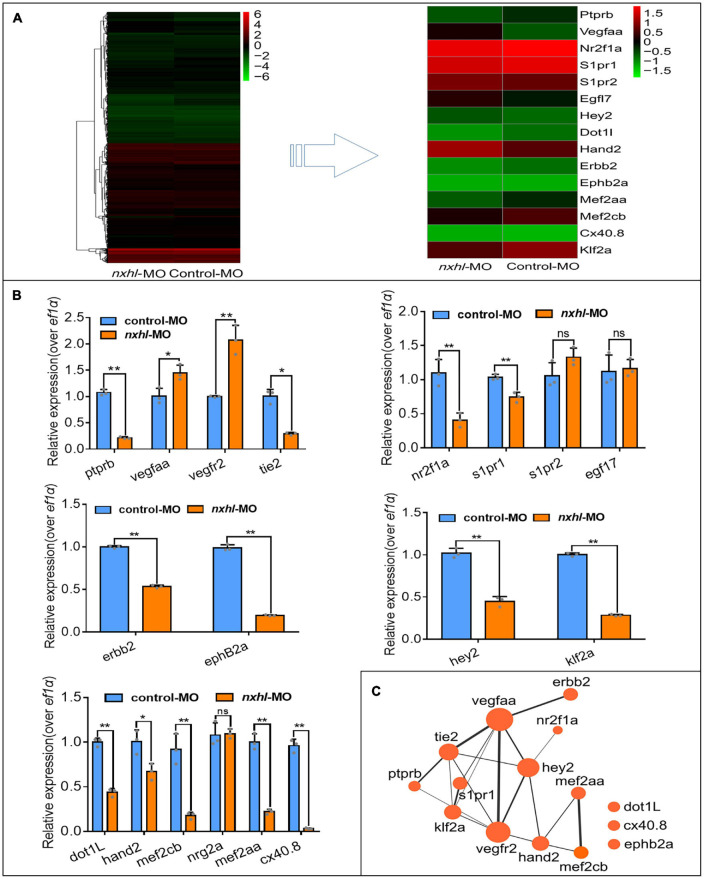
*Nxhl* modulates *ptprb* expression and angiogenic networks. **(A)** Heatmap of the 15 selected genes from zebrafishes after injection of 4 ng *nxhl*^e1i1^ MO at 3-dpf examined by RNA-seq. **(B)** Expression of genes associated with angiogenesis and/or heart development post injection of *nxhl*^e1i1^ MO 3-dpf using qPCR. The data above represent as mean ± SEM from three independent experiments. Ns, non-significant, *p < 0.05, **p < 0.001 represents statistically significant. **(C)** Networks of the genes previously reported to be associated with angiogenesis and heart development. Cytoscope V3.6.1 was used to build this network.

To investigate how *nxhl* mediates angiogenesis, we first examined transcriptome sequencing (RNA-seq) data from MO zebrafish. We found that loss of *nxhl* greatly altered the transcriptome, with 1955 downregulated and 698 upregulated genes (Supplementary Figure 5 and Supplementary Table 2). The KEGG pathways associated with angiogenesis were significantly enriched in the *nxhl*-silenced group (Supplementary Figure 6 and Supplementary Tables 2–8). We speculated that expression levels of genes linked to angiogenesis may also be significantly altered. We then screened and examined the expression of 18 genes previously documented to be closely related to heart defects and/or angiogenesis (Carra et al., 2012; Hinits et al., 2012; Ruiz-Herguido et al., 2012; Boselli et al., 2015; Pereira et al., 2016). Consistent with the RNA-seq data, we found that expression levels of 13 of these genes (*ptprb, tie2, nr2f1a, s1pr1, hey2, dot1L, hand2, erbb2, klf2a, mef2cb, mef2aa, ephB2a*, and *cx40.*8) were significantly decreased, whereas two genes (*vegfaa and vegfr2*) were sharply increased. *S1pr2, egfl7*, and *nrg2a* remained unchanged (Figures 4A,B). The arterial marker *ephB2a* and venous marker *erbb2* were decreased in *nxhl* morphants compared with the wild-type (Figure 4B). Normally, the increases of v*egfaa* and *vegfr2* are linked to enhancement of the vascular system (Koenig et al., 2016; Toselli et al., 2019). However, in our study the expression levels of both genes were increased, while others were decreased when *nxhl* was silenced. We speculated that this was a consequence of negative feedback regulation to avoid an excessive decrease in the vascular system. As previously reported, *ptprb, tie2, nr2f1a, s1pr1, vegfaa*, and *vegfr2* normally contribute to vascular development, and deletion of each leads to defects in the vascular system during embryonic development (Winderlich et al., 2009; Mendelson et al., 2013; Wu et al., 2014; Koenig et al., 2016; Toselli et al., 2019); loss of *dot1L, hand2, erbb2, mef2cb, mef2aa, ephB2a*, or *cx40.8* always results in defects in angiogenesis or heart development (Gerhart et al., 2009; Liu et al., 2010; Lazic and Scott, 2011; Nguyen et al., 2011; Hinits et al., 2012; Gemel et al., 2014; Schindler et al., 2014; Medrano and Naya, 2017). *Hey2 and klf2a* have been implicated in the regulation of both angiogenesis and heart development (Vermot et al., 2009; Gibb et al., 2018). Based on these results, we built a schematic diagram of the network, as shown in Figure 4C. This network demonstrates that silencing of *nxhl* does downregulate the key genes that are essential for heart and/or vascular development. The expression profiles of these genes can explain the *nxhl* deficiency-induced phenotypes. Notably, we found that *ptprb*, which plays essential roles in angiogenesis, was the most downregulated gene upon *nxhl* silencing (blue box in Figure 4B), indicating that it might be the target of *nxhl* regulation. Thus, we investigated whether *ptprb* has angiogenic regulatory functions similar to those of *nxhl*.

### Loss of *Ptprb* Phenocopies *Nxhl* Deficiency

**FIGURE 5:**
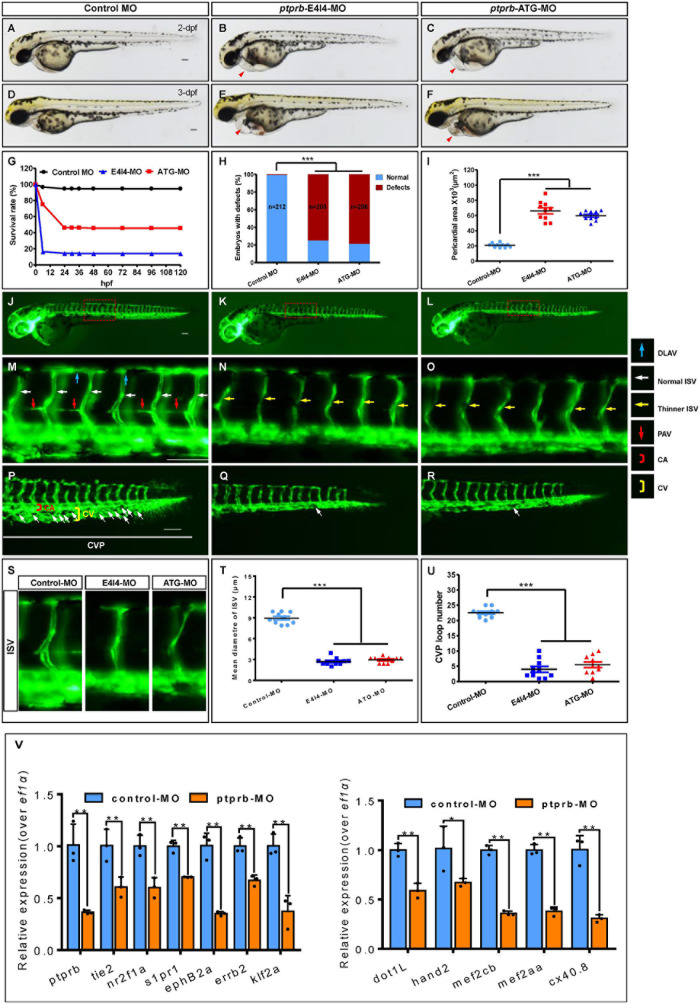
Loss of *ptprb* phenocopies *nxhl* deficiency. **(A–F)** Gross morphology at 2- and 3-dpf. Compared with control zebrafish, *ptprb* knock-down causes pericardial oedema (**B,C,E,F**, red arrowheads) and circulation defects. Heart beat and circulation in caudal vein (CV) is visible in the control fish, but is abnormal in *ptprb* morphants (Supplementary Videos 3–8). **(G)** A time-course plot of percent survival in control vs. *ptprb* morphants for 3 days. **(H)** shows the percentage of embryos with development defects. **(I)** Quantification of the pericardial area of embryos. Error bars, mean ± SEM; ***p < 0.0001 (*n* = 10; ANOVA). **(J–R)** Representative fluorescent images of *Tg(fli1a:EGFP)^y1^* embryos at 2-dpf. **(J,M)** Image of trunk regions taken at 2-dpf, with the vascular structures visualized by eGFP fluorescence and labeled ISV and DLAV showed regular development in the embryo injected with control MO. The boxed regions are shown at higher magnification in the bottom panels. **(K,L,N,O)** Compared with control MO, embryos injected with *ptprb*-MO present thinner ISVs (**N,O**, yellow arrows). In control embryos, the parachordal vessels (PAV) form normally (**M**, red arrows). Compared with control, MO knock down *ptprb* prevents the parachordal vessels (PAV) formation, the precursor to the lymphatic system. In control embryos, caudal vein plexus (CVP) were formed honeycomb-like structures at the tail around 2-dpf (**P**, white arrows). In contrast, *ptprb* knock down resulted in specific defects in caudal vein plexus (CVP) formation **(Q,R)**. **(S–U)** Quantification of the mean diameter ISVs **(S,T)** and loop formation at CVP (U) shows significantly decrease in *ptprb* morphants. Columns, mean; SEM (*n* = 10; ANOVA) ***p < 0.0001. DLAV, dorsal longitudinal anastomotic vessels; ISV, intersegmental vessel; CVP, caudal vein plexus; CA, caudal artery; CV, caudal vein; dpf, days post fertilization. Scale bar, 100 μm. **(V)** Expression of genes associated with angiogenesis (left) and heart development (right) post injection of *ptprb* MO 2-dpf using qPCR. The data represent as mean ± SEM from three independent experiments. *p < 0.05, **p < 0.001 represents statistically significant.

As described above, we noted that *ptprb* was the most downregulated gene and was closely linked to both vascular integrity and angiogenesis (Dominguez et al., 2007; Dejana et al., 2009; Mellberg et al., 2009; Hayashi et al., 2013; Braun et al., 2019; Juettner et al., 2019). To test whether there was a positive connection between *nxhl* and *ptprb*, we silenced *ptprb* in zebrafish (Supplementary Figure 7 and Supplementary Table 1). The zebrafish *ptprb* gene was targeted using a specific morpholino antisense strategy to prevent the splicing of exon 4 (E4I4-MO). ATG-MO was used as the positive control. Cleanly skipping exon 4 should frameshift the downstream sequence, leading to a premature termination codon in-frame and triggering nonsense-mediated decay of the *ptprb* transcript (Supplementary Figure 7A). We found that both *ptprb* ^e4i4^ and *ptprb*^ATG^ morphants resulted in slight pericardial edema, shortened body axis, and circulation and caudal fin defects (Figures 5A–F and Supplementary Figure 7) with significantly lower survival rate (Figures 5G,H) and smaller pericardial area of embryos (Figure 5I) at 3 dpf compared with control fish. Both *ptprb* morphants dramatically disrupted normal splicing of *ptprb* (Figures 5A–F) and decreased the survival rate but did not change *nxhl* expression, indicating high efficiency and specificity of the morpholino knockdown of *ptprb*, together with the fact that the gel band in *ptprb*-MO was obscure and degraded compared with the control MO. This confirmed that the phenotype of the *ptprb* knockdown was *nxhl*-specific rather than an off-target effect (Figures 5A–F and Supplementary Figure 7A). Consistent with this, *Ptprb* morphants also resulted in a high percentage of embryos with defects at 3 dpf (75.48%, *n* = 208 embryos in the *ptprb* MO and 0.94%, *n* = 212 embryos in controls) (Figure 5H). Moreover, heartbeat and circulation in the CV were visible in the control fish (Supplementary Videos 3, 4) but were abnormal in *ptprb*-MO-injected fish (Supplementary Videos 5–8). This confirmed that loss of *ptprb* caused the pericardial edema and circulation and caudal fin defects in the zebrafish. Compared with control MO, embryos injected with *ptprb*-MO presented thinner ISVs (Figures 5N,O, yellow arrows), and knockdown of *ptprb* prevented formation of the parachordal vessels (PAV) compared with control embryos. Furthermore, in control embryos the caudal vein plexus (CVP) formed honeycomb-like structures at the tail around 2 dpf (Figure 5P, white arrows), while *ptprb* knockdown resulted in specific defects in caudal vein plexus (CVP) formation (Figures 5J–R). Quantification of the mean diameter of ISVs (Figures 5S–T) and loop formation at CVP (Figure 5U) showed a significant decrease in *ptprb* morphants at 2 dpf (Figures 5T,U). These data are consistent with previous reports (Carra et al., 2012) and strongly suggest that loss of *ptprb* phenocopies *nxhl* deficiency. Moreover, we found that most of the 15 genes in the *ptprb*-knockdown experiment presented an expression profile similar to that caused by *nxhl* knockdown, except for vegfaa and vegfr2 (Figure 5V). To this end, we logically concluded that *ptprb* and *nxhl* act in the same signaling pathway.

### *Nxhl* Regulates *Ptprb* Through Interactions With Nucleolin

**FIGURE 6:**
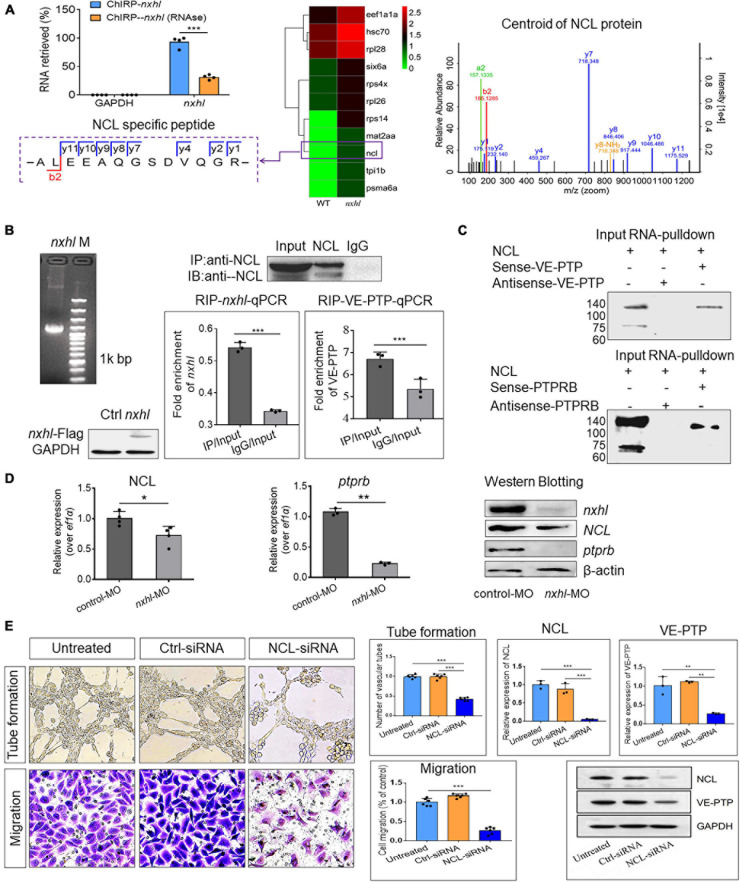
*Nxhl* regulates VE-PTP (*ptprb*) through interactions with NCL. **(A)** ChIRP-MS identification of *nxhl* RNA binding proteins using zebrafish tissues. qPCR identification of *nxhl* RNA in the eluted RNAs. Graph shows more than 90% *nxhl* RNA was retrieved, and no GAPDH was detected. Heat map shows major proteins are enriched and significantly (change fold > 2 and *p* < 0.05) retrieved by *nxhl* and control probes, analyzed by LC/MS-MS. NCL protein (purple boxed) was selected as candidate for follow-up study. The Centroid of NCL protein shows that NCL protein is pulled down and identified by LC/MS-MS. The specific peptide identifies NCL protein. **(B)** The interaction between *nxhl*, VE-PTP mRNA and NCL protein by RIP-qPCR assay. The mRNA expression of *nxhl* was determined by qPCR and Western blotting against Flag antibody and used to identify the successful expression of pcDNA3.1- Flag-*nxhl* plasmid in 293T cells. Bars show the interaction between *nxhl* mRNA and NCL protein. The interaction between VE-PTP mRNA and NCL protein is shown too, and qPCR shows the detection for VE-PTP mRNA expression in the NCL-pulled down RNA. **(C)** The interaction between *nxhl*, VE-PTP mRNA and NCL protein by pull down assay. Gels show the interaction between VE-PTP mRNA and NCL protein. Western blotting was performed to detect NCL protein in the VE-PTP-biotin probe -pulled down proteins in 293T cells. The interaction between *ptprb* mRNA and NCL protein is shown too. **(D)** Loss of *nxhl* affects the expression of NCL at both mRNA and protein levels. The mRNA expression of NCL and *ptprb* were determined by qPCR. The total NCL protein, total *nxhl* and *ptprb* protein in zebrafish tissues from knock-down group and control were detected by Western blotting using specific antibodies (details see Materials and methods). β-actin was used as internal control. **(E)** Silence of NCL inhibits angiogenesis and expression of VE-PTP. The tube formation and cell migration potential of HUVECs treated with NCL-siRNA was determined by using transwell chambers as described in the “Materials and methods” section. Scale bars, 20 μm. Representative images of cells stained in NCL-siRNA treated HUVEC cells. The expression of NCL and VE-PTP was quantified by qPCR. Protein levels of NCL and VE-PTP were examined by using Western blotting post silence of NCL. GAPDH antibody was used as internal control. The data represent as mean ± SEM from three independent experiments. *p < 0.05, **p < 0.001, ***p < 0.0001 represents statistically significant.

As shown in Figure 4, expression levels of 15 genes in the network were significantly altered in *nxhl* morphants. We supposed that these genes may be part of a regulatory network. This network presents connections between most of these genes, suggesting a cooperative regulation mechanism in heart and vascular development. Considering that *ptprb* was sharply downregulated by *nxhl* depletion and that loss of *ptprb* phenocopies *nxhl* deficiency, we next examined whether *nxhl* directly interacted with *ptprb*. Thus, we designed *nxhl* probes and conducted a chromatin isolation by RNA purification-mass spectrometer (ChIRP-MS) experiment in zebrafish to identify proteins binding to *nxhl*. Eleven proteins with a greater than twofold change were discovered (Figure 6A). Unexpectedly, *ptprb* was not found among these proteins (Figure 6A). This suggests that proteins other than *ptprb* may interact with *nxhl*. We next focused on proteins that were associated with the vascular system. NCL (Figure 6A) aroused our interest because of its molecular conservation and important functions in angiogenesis (Huang et al., 2006; Jia et al., 2017). Previous research has shown that loss of NCL in zebrafish causes edema and body axis bending (Monte et al., 2013) as well as suppression of adhesion, proliferation, and migration of HUVECs (Birmpas et al., 2012). These phenotypes are identical to the phenotypes caused by *nxhl* depletion, suggesting that NCL may be associated with *nxhl*. To ascertain whether *nxhl* interacts with NCL, we performed RNA immunoprecipitation (RIP) using NCL as the bait protein in 293T cells (Supplementary Figures 8–10) and then detected the *nxhl* RNA using quantitative polymerase chain reaction (qPCR). We found that *nxhl* RNA was significantly higher than that in an IgG control in the RNA pulled down by the NCL protein (Figure 6B), which was confirmed to be *nxhl* messenger RNA (mRNA) on reverse transcription-polymerase chain reaction (RT-PCR) amplification and RNA-seq. This indicates that the NCL protein reversely interacts with *nxhl* RNA. Therefore, these experiments indicated that *nxhl* RNA and NCL protein interact physically. However, no evidence was found regarding the interaction between *nxhl* and *ptprb*. We considered whether NCL might interact with *ptprb*, thus bridging *nxhl* and *ptprb.* Such a scenario has never been previously proposed or documented. However, one report showed that VEGF interacts with NCL (Watanabe et al., 2010). As *nxhl* acts similarly to VEGF in angiogenesis, we then supposed that NCL might also interact with *ptprb* or its human homolog VE-PTP. To test this hypothesis, we detected VE-PTP mRNA using the same RNA pulled down by the NCL protein, and we found that VE-PTP mRNA was significantly higher than that in the IgG control. The pulled-down RNA was amplified, and RNA-seq confirmed that it was VE-PTP mRNA. This confirmed that the NCL protein could also physically interact with VE-PTP mRNA (Figure 6B). We next verified this interaction in 293T cells using the VE-PTP RNA pull-down experiment in reverse; the results of Western blotting against the NCL protein supported the existence of an interaction between VE-PTP and NCL (Figure 6C). However, whether this interaction occurred between NCL and *ptprb* in zebrafish remained unclear. Therefore, we next designed a zebrafish *ptprb* gene-specific probe to pull down the proteins that interacted with *ptprb* in zebrafish. We found that the NCL protein binds strongly with *ptprb* (Figure 6C). This result indicates that the NCL protein interacts not only with VEP-PTP in 293T cells but also with *ptprb* in zebrafish.

Up to this point, we had validated that *nxhl* and NCL, and NCL and VEP-PTP (*ptprb*) interact physically. We also found that NCL was significantly downregulated at both mRNA and protein levels. We then investigated the expression of the downstream gene *ptprb* and found that loss of *nxhl* also decreased *ptprb* at both mRNA and protein levels (Figure 6D). Next, we investigated whether NCL regulates *ptprb* (VE-PTP). We thus examined the functions of NCL in angiogenesis and expression of VE-PTP by silencing of NCL in HUVECs. As shown in Figure 6E, silencing NCL significantly inhibited not only tube formation but also the cell migration of HUVECs in comparison with the controls. Notably, silencing of NCL greatly decreased the expression of VE-PTP at both mRNA and protein levels, suggesting that NCL not only interacts with VE-PTP but also regulates its expression. This highlights a regulatory role of NCL in VE-PTP expression and shows that a *nxhl–*NCL*–*VE-PTP (*ptprb*) signaling pathway is logical and reasonable in angiogenesis. These results suggest that silencing of *nxhl* leads to angiogenesis defects owing to the downregulation of both NCL and *ptprb* via the interactions of *nxhl–*NCL and NCL*–ptprb*, which consequently mediate the angiogenesis-linked landmark gene network.

### *Nxhl* Attenuates Tumor Invasion and Proteins Associated With Angiogenesis and Epithelial-Mesenchymal Transition

**FIGURE 7:**
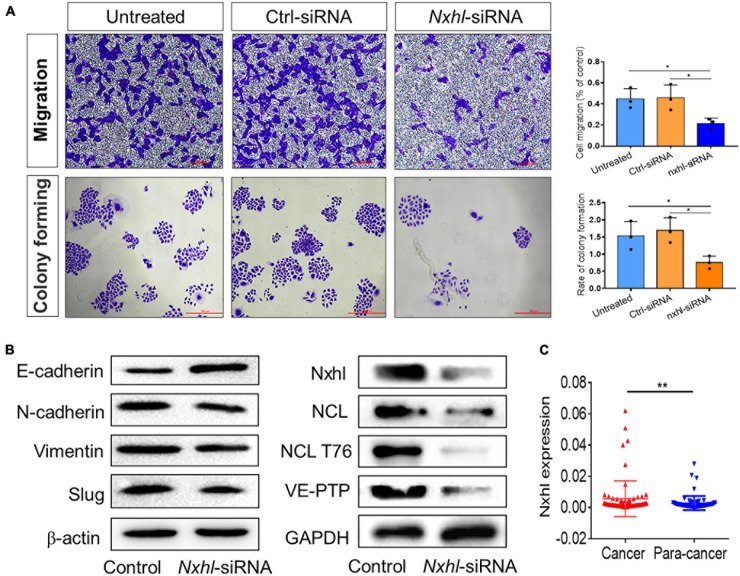
*Nxhl* attenuates tumor invasion and proteins associated with angiogenesis and EMT. **(A)** Silence of *nxhl* (homolog of Harbi1) inhibits migration and EMT of HCC cells. Representative images of migrated cells stained with crystal violet and inhibition of migration in *nxhl*-siRNA treated HepG2 cell (up row). Representative images of colony formation cells stained with crystal violet and inhibition of colony forming in *nxhl*-siRNA treated HepG2 cell (down row). **(B)** Silence of *nxhl* inhibits epithelial-mesenchymal transition of HepG2 cells. Four EMT marker proteins, NCL, NCL T76 and VE-PTP were detected using Western blotting method in control and siRNA-treated HepG2 cells. **(C)** *nxhl* (Harbi1) expression in 72 HCC tissues and para-cancer tissues. qPCR was used to determine the human homolog of *nxhl* (Harbi1) expression levels. The data above represent as mean ± SEM from three independent experiments. *p < 0.05, **p < 0.001 represents statistically significant.

Because targeting angiogenesis has great potential in anti-tumor or anti-cancer therapy (Castet et al., 2019; Qin et al., 2019), we next investigated whether this anti-angiogenesis function affects the angiogenesis of cancer cells. HepG2 (hepatocellular carcinoma, HCC), A549 (non-small cell lung cancer, NSCLC), and HCT116 (colon carcinoma, Colo) cells were transferred with *nxhl*-siRNA, and their supernatants were subsequently incubated with HUVECs. We observed that the abilities of tube formation in all cancer cells were significantly inhibited compared with controls (Figure 7 and Supplementary Figures 11B,C), suggesting that silencing of *nxhl* suppresses angiogenesis in cancer cells. We further silenced *nxhl* in HepG2 cell lines and found that loss of *nxhl* not only significantly inhibited the proliferation, migration, and colony formation of HCC cells but also suppressed the expression of EMT marker genes, suggesting that silencing of *nxhl* inhibits the EMT process of cancer cells (Figure 7B and Supplementary Figure 11C). Moreover, silence of *nxhl* also inhibited NCL, NCL T76, and VE-PTP (*ptprb*) in cancer cells, identical to the signaling pathway induced by its homolog *nxhl* (Figure 7B). We then predicted whether *nxhl* is linked to HCC progression based on data in the cancer genome atlas (TCGA). We found that expression of *nxhl* in HCC tissues was significantly higher than that in normal tissues (Supplementary Figure 11D). We next tested this in tissue samples from 72 patients with HCC using qRT-PCR and found that the clinical test results were consistent with the TCGA predictions (Figure 7C and Supplementary Figure 11D). This function of *nxhl* has never been reported in cancer studies (Kapitonov and Jurka, 2004; Sinzelle et al., 2008; Smith et al., 2012). Our data indicate that nxhl has a role in the angiogenesis process via ECs and is a potential anti-cancer therapeutic target.

### *Nxhl* Controls Angiogenesis by Targeting Vascular Endothelial Protein Tyrosine Phosphatase (*ptprb*) and Linking Angiogenesis Regulatory Genes

**FIGURE 8:**
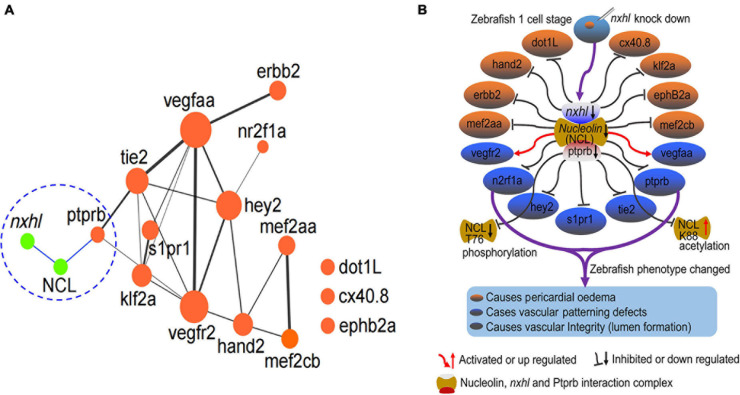
*Nxhl* controls angiogenesis by targeting VE-PTP (*ptprb*)-related angiogenic genes. **(A)** Schematic model illustrating the mechanism of *nxhl* in zebrafish angiogenesis and heart development. The interactions between *nxhl* mRNA and NCL protein, NCL protein and *ptprb* mRNA are new-found interactions in this study. It connects with the keystone network of angiogenesis and heart development. **(B)** Possible mechanism of *nxhl* in zebrafish angiogenesis and heart development. Knockdown of *nxhl* may downregulate the *nxhl*-NCL-*ptprb* complex, subsequently regulate the proteins associated with angiogenesis and heart development, and finally result in heart pericardial edema, vascular patterning and integrity defects.

Based on above results, we concluded that *nxhl* controls angiogenesis through *nxhl–*NCL*–*VE-PTP (*ptprb*)-linked angiogenesis regulatory genes. These findings are the first to uncover the existence of upstream regulatory genes of VE-PTP (*ptprb*). We then built a new schematic diagram based on the network in Figure 4C that shows the novel *nxhl–*NCL*–*VE-PTP (*ptprb*) signaling links to the keystone angiogenesis genes (Figure 8A vs. Figure 4C). We also created a schematic diagram to describe the possible mechanism underlying *nxhl-*induced phenotypes (Figure 8B). Knockdown of *nxhl* significantly and broadly downregulated angiogenesis-associated landmark genes, including d*ot1L, hand2, erbb2, mef2aa, n2rf1a, hey2, s1pr1, tie2, ptprb, meff2cb, ephB2a, klf2a*, and *cx40.8*, through the *nxhl–*NCL–VE-PTP (*ptprb*) pathway, and *vegfr2* and *vegfaa* negative feedback control this downregulation. Moreover, loss of *nxhl* increased the phosphorylation of NCL (T76), indicating that *nxhl* may control angiogenesis by impacting NCL posttranslational modification to regulate downstream VE-PTP signaling pathways. This highlights the crucial role of *nxhl* in angiogenesis via a hitherto unreported *nxhl–*NCL–VE-PTP (*ptprb*) pathway that extends the upstream regulatory members of the keystone gene VE-PTP (*ptprb*). We conclude that *nxhl* controls angiogenesis by targeting VE-PTP (*ptprb*) through interaction with NCL and by linking vascular keystone regulatory genes. Given the extreme importance of angiogenesis and the broad connections with landmark genes, we believe that the identification of this novel signaling pathway to be of considerable importance for the study of angiogenesis development and treatment of cancers.

## Discussion

Previous studies have shown that VE-PTP is a key player in the regulation of angiogenesis and the EC adherens junctions (Baumer et al., 2006; Winderlich et al., 2009; Hayashi et al., 2013; Monaghan-Benson and Burridge, 2013) as well as a potential therapeutic target for angiogenesis-dependent diseases (Wilke et al., 2014; Ferrara and Adamis, 2016). VE-PTP binds to several proteins, including Tie2, VEGFR2, VE-cadherin, and FGD5, that mediate angiogenic signaling pathways (Christian et al., 2003; Fogal et al., 2009; Koutsioumpa and Papadimitriou, 2014; Campochiaro et al., 2015; Vestweber, 2015). In the present study, we identified a novel zebrafish gene, *nxhl*, that controls angiogenesis *in vivo* and *in vitro* by targeting VE-PTP through interaction with NCL. We showed that *nxhl* physically binds to NCL, a protein that interacts with VE-PTP and thereby controls angiogenesis. Moreover, *nxhl* exhibits promising potential for the treatment of cancers. We described a novel *nxhl–*NCL*–*VE-PTP signaling pathway for angiogenesis regulation.

Anti-angiogenic drugs have been a focus of study, and many inhibitors of angiogenesis are currently used as monotherapy or in combination with chemotherapy or cytokine treatment (Ebos and Kerbel, 2011). Previous studies have highlighted the value of VE-PTP in anti-angiogenic agents (Huang et al., 2006; Destouches et al., 2012; Gilles et al., 2016; Quiroz-Mercado et al., 2016). However, the upstream regulation mechanisms of VE-PTP are unknown. In this study, we identified *nxhl* as a novel powerful upstream regulator of VE-PTP based on the finding that *nxhl* not only sharply decreases expression of VE-PTP and other key angiogenic genes but also that its deletion causes angiogenesis phenotypes that resemble VE-PTP deficiency. Moreover, *nxhl* controls angiogenesis via EC migration and tube formation, consistent with the angiogenic characteristics of VE-PTP in EC adhesion and integrity (Nawroth et al., 2002; Nottebaum et al., 2008; Vestweber, 2015), confirming that its angiogenesis controlling function acts via ECs. This was also strongly supported by our finding that silencing of the highly conserved human homolog of *nxhl* not only inhibits HUVEC migration and tube formation but also suppresses the migration and invasion of cancer cell lines by inhibiting ECs. All these data suggest that *nxhl* is a powerful upstream angiogenesis controller targeting VE-PTP.

The effects of *nxhl* controlling angiogenesis depend on its binding with NCL, which simultaneously bridges *nxhl* and VE-PTP. To the best of our knowledge, this is the first description of the interactions between *nxhl* and NCL, and NCL and VE-PTP, uncovering a novel angiogenesis signaling complex upstream of VE-PTP. NCL is expressed broadly in all cells in a proliferation-dependent manner (Roussel and Hernandez-Verdun, 1994) and in nearly all cell compartments. Like VEP-TP, NCL is also associated with cancer and other angiogenic diseases. However, this function is more likely related to the cell surface NCL rather than those in other compartments. The cell surface NCL is clustered and highly expressed in ECs of angiogenic blood vessels during angiogenesis (Hovanessian et al., 2000; Christian et al., 2003; Fogal et al., 2009), suggesting that NCL functions as an angiogenic gene. Previous studies have defined NCL as both a prognostic marker and a therapeutic target (Srivastava et al., 1995; Schwab and Dreyer, 1997; Yelon et al., 2000; Di Segni et al., 2008; Wolfson et al., 2018), highlighting its potential value in the development of anti-angiogenic drugs (Huang et al., 2006; Jia et al., 2017). In this study, we identified the direct interactions between *nxhl* and NCL, and NCL and VE-PTP (*ptprb*) in both zebrafish and 293T cells, although we have not yet determined which subset of NCL (surface, nucleolar, or cytoplasmic NCL) participates in this interaction. Importantly, we demonstrated that silencing of NCL inhibits angiogenesis of HUVECs and expression of VE-PTP at both mRNA and protein levels. This further supports the hypothesis that NCL plays a key role in angiogenesis by directly controlling downstream VE-PTP. Moreover, deletion of *nxhl* caused a significant decrease in NCL, suggesting that *nxhl* significantly affects and regulates NCL. Previous studies have suggested that NCL phosphorylation status heavily affects its cellular compartmentalization (Schwab and Dreyer, 1997). This promotes EGFR phosphorylation, dimerization, and cell growth (Di Segni et al., 2008; Farin et al., 2011) and also promotes HER2 (namely, Erbb2) phosphorylation and subsequent MAPK/ERK pathway activation (Wolfson et al., 2016). Clinically, combination treatment with NCL and HER2 inhibitors exhibits superior efficacy compared with single treatment in the invasion capacity of breast cancer cells (Wolfson et al., 2018). Because EGFR and HER2 are closely associated with angiogenesis, we consider that *nxhl* may control angiogenesis by affecting NCL phosphorylation, thereby regulating downstream EGFR and HER2 signaling pathways. Notably, in our study the expression of Erbb2 was significantly affected by *nxhl* knockdown, partially supporting this point of view. However, this question needs to be investigated in future work. Taken together, our data enable us to conclude that *nxhl* regulates angiogenesis via the *nxhl*–NCL–VE-PTP (*ptprb*) pathway.

The strong effect of *nxhl* in controlling angiogenesis also relies on the effects of several other crucial downstream angiogenic genes (Tie2, VEGFaa, VEGFR2, S1pr1, and Hey2) that are broadly associated with VE-PTP signaling (Figures 4, 7). What must be stressed is that the expression of these genes can explain the phenotypes induced by *nxhl* deficiency. These genes all play irreplaceable roles in multiple aspects of angiogenesis during development. For example, Hand2 is vital in heart development in the zebrafish and mice (Srivastava et al., 1995; Yelon et al., 2000). It has been identified as a specifier of outflow tract cells in the mouse (de Soysa et al., 2019). Hey2 mediates the dynamics of cardiac progenitor cells in addition to the zebrafish heart (Gibb et al., 2018). It has been identified as a component of the NKX2-5 cardiac transcriptional network regulating the early stages of human heart development (Anderson et al., 2018). Dot1L (Nguyen et al., 2011), Mef2aa (Medrano and Naya, 2017), Mef2cb (Lazic and Scott, 2011), Erbb2 (Liu et al., 2010), Klf2a (Vermot et al., 2009), and EphB2a (Gerety and Anderson, 2002) also play key roles in the growth of the chamber, cardiomyocyte differentiation, valvulogenesis, and myocardial trabeculation during heart development. Importantly, heart and vascular development are always linked. Previous studies have shown that silencing of zebrafish S1pr1 not only leads to global and pericardial edema, a lack of blood circulation, and reduced vascularization in ISVs and CVPs (Mendelson et al., 2013) but also regulates endothelial barrier integrity via the S1pr1/VE–cadherin/EphB4a pathway (Tobia et al., 2012). Similar phenotypes can be induced by the knockdown of Nr2fla in zebrafish (Wu et al., 2014). Moreover, mutation of Nr2f1a results in smaller atria owing to a specific reduction in the atrial cardiomyocyte number and an increase in the rate of atrial cardiomyocyte differentiation (Duong et al., 2018). Another key gene, Tie2, is essentially required for ISV growth, sprouting, migration, and proliferation of tip cells and acts in coordination with VEGF signaling (Li et al., 2014). Loss of Tie2 leads to death at E10.5 owing to vessel remodeling defects and lack of trabeculation (Sato et al., 1995). Notably, the Ang-Tie2 system is indispensable for vascular and lymphatic development (Eklund et al., 2017). The anti-angiogenic effects of the VE-PTP inhibitor AKB-9778 likely rely on the Ang-Tie2 pathway (Nottebaum et al., 2008). In our study, *nxhl* deletion led to a significant decrease in Tie2, suggesting that it regulates not only VE-PTP but also the Ang-Tie2 system, which cross-talks with VE-PTP. From this point of view, *nxhl* is a multifunctional regulator of the angiogenesis process. This explains our findings that the phenotypes induced by *nxhl* knockdown mostly resemble the phenotypes caused by deletion of the genes associated with VE-PTP. Given the extreme importance of these genes in angiogenesis during development, we consider the phenotypes caused by *nxhl* morphants to be direct or indirect consequences of the downregulation of VE-PTP and these key genes. We believe that the present novel *nxhl*–NCL–VE-PTP signaling pathway is crucial in vertebrate angiogenesis regulation and anti-angiogenic diseases. This is supported by our findings that silencing of *nxhl* not only inhibits the proliferation, invasion, migration, and colony formation of HCC cells but also suppresses the EMT process as well as NCL, NCL T76, and VE-PTP (*ptprb*) in cancer cells. This again indicates that *nxhl* plays a significant role in regulation of angiogenesis, implying its value in anti-angiogenic drug exploration.

In this study, we demonstrated that a novel gene, *nxhl*, controls angiogenesis by targeting VE-PTP through interaction with NCL. Furthermore, we have elucidated some of the crucial downstream pathways that may be implicated in regulation of angiogenesis. This study is the first to reveal a new *nxhl*–NCL–VE-PTP signaling pathway governing vertebrate angiogenesis and its potential as a therapeutic target for cancer treatment.

## Data Availability Statement

The authors declare that all data reported in this study are fully and freely available from the date of publication. RNA-Seq data of the embryo are available under BioProject PRJNA574895. Transcriptome (Illumina) data of *nxhl* silence are available in the Sequence Read Archive (SRA) with accession numbers SRR10199007 and SRR10199008 under BioProject PRJNA573544.

## Ethics Statement

The animal study was reviewed and approved by the SRCMO IACUC (No. 2018-0019).

## Author Contributions

HLL and XHC designed the scientific objectives and oversaw the project. HLL, XHC, YDZ, YFD, and YL discussed the primary ideas of the manuscript. YDZ, YL, LQL, ZAS, YLY, and PFF collected samples for sequencing DNA and RNA. HLL and YDZ performed RNA-seq analysis and performed functional assay of zebrafish *nxhl* gene. YDZ, HLL, and YFD prepared the Supplementary Data and Method. XHC, YFD, and HLL prepared the draft manuscript with input from all other authors. HLL, XHC, YFD, and YL discussed and revised the manuscript. All authors contributed to the article and approved the submitted version.

## Conflict of Interest

The authors declare that the research was conducted in the absence of any commercial or financial relationships that could be construed as a potential conflict of interest.

## Publisher’s Note

All claims expressed in this article are solely those of the authors and do not necessarily represent those of their affiliated organizations, or those of the publisher, the editors and the reviewers. Any product that may be evaluated in this article, or claim that may be made by its manufacturer, is not guaranteed or endorsed by the publisher.

## Footnotes

1
http://www.gene-tools.com/

2
http://rsbweb.nih.gov/ij/

3
http://www.bioinformatics.babraham.ac.uk/projects/fastqc/

4
http://www.usadellab.org/cms/?page=trimmomatic

5
http://deweylab.github.io/RSEM/

6
https://github.com/skygenomics/CSBB-v1.0

7
https://toppgene.cchmc.org/

## Supplementary Material

Supplementary Figure 1 Expression profile of introns in Golden Pompano embryo development. **(A)** 19 embryo development stages were collected and transcriptome-sequenced. The FPKM of introns were used to draw the heatmap. A boundary lines between OSP-MGS and LGS-YAPS stages. **(B)** GO cluster of the differentially expressed genes. **(C)** KEGG cluster of the differentially expressed genes. Numbers of 1–57 below the heatmap represent embryo samples. Every three numbers represent one embryo stage. For example, number 1–3 represents stage OSP, number 55–57 represents stage YAPS.

Supplementary Figure 2 Confirmation of *nxhl* gene knockdown. **(A)** Schematic map of *nxhl* gene and the MO design and micro-injection strategy. 4 ng morphants per fish embryo was injected. **(B)** The gel electrophoresis of PCR products of *nxhl* gene in control and *nxhl-*MO groups. Compared with control MO, *nxhl* MO shows quite weak expression of *nxhl* gene. **(C)** Expression profile of *nxhl* at 6 and 3 dpf in zebrafish embryo. hpf, hours post fertilization; dpf, days post fertilization.

Supplementary Figure 3 Phenotypes of *nxhl* zebrafish morphants. 4 ng morphants per fish embryo was injected. **(A)** Gross morphology at 3-dpf. Compared with control MO, knock down *nxhl* present pericardial edema (red arrow) and caudal fin defects (blue arrow). **(B)** Representative bright field and fluorescent images of *Tg(fli1a:EGFP)^y1^* embryos at 2-dpf after injection of *nxhl* morphants.

Supplementary Figure 4 Knockdown efficiency of the siRNAs. Trails for the choose of siRNAs before the formal experiments were performed. Three siRNAs targeting the *nxhl* gene were designed and tested for the knockdown efficiency. The siRNA-2 with the highest knockdown efficiency (about 70%) was chosen to perform the subsequent experiments.

Supplementary Figure 5 Gene expression profile post knockdown of *nxhl* comparing with control. Scatter of the differential expressed genes after injection of *nxhl* morphants.

Supplementary Figure 6 KEGG enrichment of genes differentially expressed after knockdown of *nxhl* gene comparing with control.

Supplementary Figure 7 Phenotypes of *ptprb* zebrafish morphants. 4 ng morphants per fish embryo was injected. **(A)** Effectiveness of *ptprb* knockdown was confirmed by RT-PCR and Sanger sequencing. The zebrafish *ptprb* gene was targeted by specific morpholino antisense to prevent proper splicing of exon 4 (E4I4-MO). Primers 3F and 5R interrogate the presence of wild type (non-mutant) transcripts or those in which exon 4 has been skipped. Below the diagram is a schematic depiction of the exon 4-skipped transcript in the E4I4-MO–injected embryos (124 bp) as compared with control embryos (388 bp). RT-PCR of *ptprb* transcript from control and E4I4-MO morpholino- injected embryos 3 days after fertilization, demonstrating skipping of exon 4. Sanger sequencing of both the wild type band and the exon 4-skipped band validating the wild type sequence and the exon 4-skipped sequence. **(B)** Gross morphology at 2-dpf. Compared with control MO, knock down *ptprb* present pericardial oedema (red arrow). **(C)** Representative bright field and fluorescent images of *Tg(fli1a:EGFP)^y1^* embryos at 2-dpf after injection of *ptprb* morphants.

Supplementary Figure 8 Identification of pcDNA3.1-*nxhl* expression plasmid. **(A)** PCR amplification of *nxhl* gene (1,267 bp, including enzyme cutting sites NheI and KpnI, and protective bases). **(B)** Four monoclones were selected by PCR and used for sequencing identification. **(C)** Sequencing result of *nxhl* gene. The green underline is the fragment inserted into the vector, the vector sequence is on both sides. The red underline is the sequencing result of enzyme cutting site, which shows that the target sequence has been correctly constructed into the vector.

Supplementary Figure 9 Identification of pcDNA3.1-*nxhl*-Flag expression plasmid. **(A)** PCR amplification of *nxhl*-Flag gene (1,306 bp, including enzyme cutting sites NheI and KpnI, and protective bases) based on pcDNA3.1-*nxhl* constructs. **(B)** Sequencing result of *nxhl*-Flag fragment. The green underline is the fragment inserted into the vector, which shows that the target sequence has been correctly constructed into the vector.

Supplementary Figure 10 SDS-PAGE electrophoresis of *ptprb* and *nxhl* protein. *Ptprb* and *nxhl* protein was expressed in pet-b2m Rosetta expression system with kanamycin resistance, respectively. *Ptprb* (33 kDa) and *Nxhl* (62 kDa) was successfully expressed and the purity of the recombinant protein was 85%. The purified protein was used to immunize rabbits to produce antibody, respectively. M, protein marker. 1, whole bacterial protein. 2, pet-b2m-*Nxhl* protein induced by IPTG. 3 and 4 pet-b2m-*ptprb* protein induced by IPTG.

Supplementary Figure 11 Silencing of *nxhl* not only decreases the angiogenesis of cancer cell lines but inhibits migration and EMT of HCC cells. **(A)** Silence of *nxhl* (Harbi1) gene inhibits angiogenesis of two cancer cell lines in vitro. HUVEC cells were plated on matrigel-coated plates at a density of 1.5 × 10^5^ cells/well and incubated for 24 h before the cell culture supernatant of *nxhl*-siRNA treated non-small-cell lung carcinoma (A549) and colon carcinoma (HCT116) cell lines was added, respectively. Representative images of tube formation in *nxhl*-siRNA treated A549 cells. Representative images of tube formation in *nxhl*-siRNA treated HCT116 cells. The data represent as mean ± SEM from three independent experiments. Scale bars, 100 μm. Images were taken 24 h after addition of the supernatant. Tube networks were quantified using the ImageJ software and expressed as branches. **(B)** Silence of *nxhl* (Harbi1) inhibits migration and EMT of HCC cells. Representative images of migrated cells stained with crystal violet and inhibition of migration in *nxhl*-siRNA treated SMMC7721 cell. Representative images of colony formation cells stained with crystal violet and inhibition of colony forming in *nxhl*-siRNA treated SMMC7721 cell. **(C)** Silence of *nxhl* (Harbi1) inhibits SMMC7721 cell growth. MTT method was used to test the cell proliferation. **(D)** *Nxhl* (Harbi1) gene expression in HCC samples based on the TCGA data. The data represent as mean ± SEM from three independent experiments. ^∗^*p* < 0.05, ^∗∗^*p* < 0.001, ^∗∗∗^*p* < 0.0001 represents statistically significant.

Supplementary Table 1 Primers and probes used in this study.

Supplementary Table 2 Annotation of the differentially expressed genes.

Supplementary Table 3 Differentially expression analysis between *nxhl* MO and Control MO.

Supplementary Table 4 Go analysis of the down-regulated genes between *nxhl* MO and Control MO.

Supplementary Table 5 Go analysis of the up-regulated genes between *nxhl* MO and Control MO.

Supplementary Table 6 KEGG pathway analysis.

Supplementary Table 7 All genes involved in KEGG pathway.

Supplementary Table 8 GO enrichment of genes involved in blood vessel morphogenesis and their differential expression value.

Supplementary Video 1 Heart beat. *Nxhl* was silenced by using morpholino knockdown technique and the CV of zebrafish was observed under microscope. The video showed the heart beat in splice-blocking and ATG morpholino group, respectively, with comparison to control group.

Supplementary Video 2 Caudal vein (CV) circulation. *Nxhl* was silenced by using morpholino knockdown technique and the CV of zebrafish was observed under microscope. The video showed the CV circulation in splice-blocking and ATG morpholino group, respectively, with comparison to control group.

Supplementary Video 3 Heart beat focused. *Nxhl* was silenced by using morpholino knockdown technique and the CV of zebrafish was observed under microscope. The video showed the heart beat in morpholino group with comparison to control group.

Supplementary Video 4 CV circulation focused. *Nxhl* was silenced by using morpholino knockdown technique and the CV of zebrafish was observed under microscope. The video showed the CV circulation in morpholino group with comparison to control group.

Supplementary Video 5 Heart beat. *Ptprb* was silenced by using morpholino knockdown technique and the CV of zebrafish was observed under microscope. The video showed the heart beat in ATG morpholino group with comparison to control group.

Supplementary Video 6 CV circulation. *Ptprb* was silenced by using morpholino knockdown technique and the CV of zebrafish was observed under microscope. The video showed the CV circulation in ATG morpholino group with comparison to control group.

Supplementary Video 7 CV circulation. *Ptprb* was silenced by using morpholino knockdown technique and the CV of zebrafish was observed under microscope. The video showed the CV circulation in splice-blocking morpholino group with comparison to control group.

Supplementary Video 8 CV circulation. *Ptprb* was silenced by using morpholino knockdown technique and the CV of zebrafish was observed under microscope. The video showed the CV circulation in ATG-blocking morpholino group with comparison to control group.
