# Supplementary material for: Nxhl Controls Angiogenesis by Targeting VE-PTP Through Interaction With Nucleolin
Source: Front Cell Dev Biol. 2021 Oct 11;9:728821. doi: 10.3389/fcell.2021.728821 (PMC8558974; doi:10.3389/fcell.2021.728821)
Supplement: Supplementary Table 1 — Primers and probes used in this study. [file Data_Sheet_3.zip › supplementry tables (frionter)/Supplementary Tables (Table S1).docx]

**Table S1. Primers and probes used in this study.**

| Gene name | Primers (5’to 3’) | Tm (℃) | |
| --- | --- | --- | --- |
| QPCR | | | |
| Ze-ptprb | FOR:GGTCGAACTGGGACTTTCATAG  REV:CCGCACACACTGATGGATATAA | | 58 |
| Ze-VEGFaa | FOR: AAAAGAGTGCGTGCAAGACC  REV:GACGTTTCGTGTCTCTGTCG | | 59 |
| Ze-VEGFR2 | FOR:GAGAGCCAGCGACTCACCGCAACAC  REV:GTTCGCTCGATCATCATCTTGGC | | 58 |
| Ze-tie2 | FOR:GCCGTCAAGAGGATGAAAGA  REV:CCCAGCAGGTGTATGATGTT | | 58 |
| Ze-nr2f1a | FOR:AAGCTGACCGAGAGCAACTC  REV:CGCCGCCACCCATGA | | 58 |
| Ze-s1pr1 | FOR:ATCACCAGCCGAGAAACCTAC  REV:GTGAGCGCACTTGTTTCCAA | | 60 |
| Ze-s1pr2 | FOR:GGTCAGAAGGAACGACACCA  REV:CCACCAACTGCTTCCGTTTG | | 60 |
| Ze-egfl7 | FOR:CTCTCACGACCTCTCCAACG  REV:GCGGGAAAGACCTGATGGAC | | 59 |
| Ze-hey2 | FOR:CGGCTTCCGGGAGTGTCTGACT  REV:TCCCCACGGTCGGTATGGTTTA | | 58 |
| Ze-dot1L | FOR: CATGATGCTGCACACGAAATC  REV:GCATACTCTCGAAGCTCTTGG | | 59 |
| Ze-hand2 | FOR: CAGACGCCAAAGAAGAAAGGCGAA  REV:CATTGCTTCAGCTCCAATGCCCAA | | 59 |
| Ze-erbb2 | FOR:GACTTCACTGCTCCACCCAA  REV:CCCAACAACCTGAATCCCCA | | 60 |
| Ze-klf2a | FOR:CCGTCTATTTCCACATTTTCG  REV:TCCAGTTCATCCTTCCACCT | | 60 |
| Ze-mef2cb | FOR:CACACGGATTATGGATGAACG  REV:TCCTTTGACTCTGGGCTGTGG | | 58 |
| Ze-mef2aa | FOR:GGATAAGCAGTTCCCAGTCG  REV:CTGATGGACTCTCCCTCTCG | | 61 |
| Ze-nrg2a | FOR:GCCGGCAACTGAGAGAGTAAT  REV:CGCTGACACATACCTGTGGG | | 60 |
| Ze-nxhl | FOR:CGACGACGTAACCGCCCATAA  REV:AGTTGCCGTCCGTCTTCTCATCA | | 58 |
| Ze-ephB2a | FOR:ACCCTACCAGTTACCCTCCC  REV:CCATCTCCCTTATCTTCCCCA | | 60 |
| Ze-CX40.8 | FOR: ATTACAATGACTGCTCGGCCC  REV:TGCTCATTAGCCTGCTTGTCG | | 58 |
| Ze-NCL | FOR: CGATGGCGAGGGTTTCA  REV:TTCTTGTTTTCCTGCGAGTT | | 60 |
| Ze-ef1α | FOR:GGAAATTCGAGACCAGCAAATAC  REV:GATACCAGCCTCAAACTCACC | | 60 |
| Ze-eif4g2α | FOR:GAGATGTATGCCACTGATGAT  REV:GCGCAGTAACATTCCTTTAG | | 59 |
| Ze-18S rRNA | FOR:AATTGCGCGTCAACTTGTGAACTG  REV:AGGAGTGGTCCAGGAACGAGTATC | | 60 |
| GP-T28534 | FOR:TGATTGGCTTGCCTTCCTGTTCG  REV:AAGCGACTCTTGTTCAGCCTCTTG | | 60 |
| GP-T28322 | FOR:GTTCTGGCTGGAGTAGTGGTTGTG  REV:AGCAATCTGGCAGAGACGCAATG | | 59 |
| GP-E1646 | FOR:ACTGGCGGTAATGTTGCTGAGATG  REV:GCTGGTGTCCTGTGCGTAATCG | | 60 |
| GP-T22774 | FOR:AAGGTGTCCACATTGTCGGCAAC  REV:GGTGGTGGTGGCGGCATATTG | | 59 |
| GP-T22775 | FOR:GGTGGTGGTGGCGGCATATTG  REV:AAGGTGTCCACATTGTCGGCAAC | | 60 |
| GP-E6367 | FOR:ACGGATGTCTCCAGGAGATGCC  REV:TGCTGCTGAAGTCTGTGCGATG | | 60 |
| GP-E22395 | FOR:ACCGTCGCTGACCGATTACAATG  REV:TTGTCCTCTGCCAGCTCCTGTAG | | 59 |
| GP-E22044 | FOR:GCCAAGCCGAGCGTCACTTAC  REV:CACAAGCAGCACTCCGATCACC | | 58 |
| GP-EVM0000103 | FOR:CCTACGACTACTCCTCCATCAT  REV:GCAGCCATAGAGCGTGTTG | | 60 |
| GP-EVM0000113 | FOR:AACTACACCAACCAGGACTACC  REV:CACGCCATACATACACCACAC | | 60 |
| GP-EVM0000361 | FOR:ATGAAGGAGGCGGTGAGGA  REV:TCTGCGATACGAGTCTTGATGA | | 59 |
| GP-EVM0000779 | FOR:CCTGAATCGGAGCCTGAAGT  REV:GCTGTAACGGCACTGTGAG | | 61 |
| GP-EVM0000810 | FOR:TCAGTCCACATCAGATTCAAGA  REV:TAACTATCAGCACCAGCAGAG | | 58 |
| GP-EVM0000837 | FOR:TTCGCTGCTTCCTTCTCCAT  REV:CCAGGTAGTCTCCGTTAGTGTA | | 59 |
| GP-EVM0001034 | FOR:CGCAGAGATGTGGAGCAGAT  REV:TGGGAGTAAGTGTGGGATTTGA | | 60 |
| GP-EVM0001183 | FOR:CGATTACCGACTTCAGCAGAG  REV:CCGAATCCTTCCAGAGTGTTC | | 61 |
| GP-EVM0001568 | FOR:CCAAGTCTAAGAACGGAGGAAG  REV:CTCAGTCTCGCATACATAACCA | | 60 |
| GP-nxh | FOR:CAAGGTGTCCATCTCTGGCTGTG  REV:TGTAGTTGTAGGTGCGTTGCTCAG | | 59 |
| Hu-Harbi1 | FOR:CAGGCGTCTATGAGTCGTT  REV:AGAGGTCTTCAGCATTTGG | | 60 |
| Hu-VE-PTP | FOR:GCGGACCAGGATTCCCTCTA  REV:AACTCCCGGATGGTCC | | 60 |
| Hu-NCL | FOR: GCTGCAAAAGTTGTTCCTGTGA  REV: TTGAAAGCCGTAGTCGGTTCT | | 59 |
| Hu-VEGFR2 | FOR: GCCAGTCTTCTAGGCATATCC  REV: CTCCCCAGGTACTGCTACTT | | 60 |
| Hu-Tie2 | FOR: TTATGAGAGGCCATCATTTG  REV: CTCATAAAGCGTGGTATTCA | | 60 |
| Hu-β-actin | FOR:AGCGAGCATCCCCCAAAGTT  REV:GGGCACGAAGGCTCATCATT | | 59 |
| Hu-18S | FOR: CCTGGATACCGCAGCTAGGA  REV: GCGGCGCAATACGAATGCCCC | | 59 |
| Hu-Slug | FOR:AACAGTATGTGCCTTGGGGG  REV:AAAAGGCACTTGGAAGGGGT | | 59 |
| Hu-E-cadherin | FOR:TGAAAACAGCAAAGGGCTTGGA  REV:GCAGTGTCTCTCCAAATCCGA | | 60 |
| Hu- N-cadherin | FOR:GTGCATGAAGGACAGCCTCT  REV:GCCACTTGCCACTTTTCCTG | | 60 |
| Hu- vimentin | FOR:TCCGCACATTCGAGCAAAGA  REV:TGAGGGCTCCTAGCGGTTTA | | 60 |
| Hu-β-actin | FOR:AGCGAGCATCCCCCAAAGTT  REV:GGGCACGAAGGCTCATCATT | | 59 |
| SiRNA | | | |
| Si-hu-Harbi1  Si-hu-NCL | AUGUUAUAGCGAUAUUCUGCUTT  CAGAAUAUCGCUAUAACAUGGTT  GGAACUCACUGGUUUGAAA dTdT  UUUCAAACCAGUGAGUUCC dTdT | |  |
| Morpholino Knockdown | | | |
| Control-MO  (Ze-nxhl) | 5′- CCTCTTACCTCAGTTACAATTTATA -3′ | |  |
| Ze-nxhl-e1i1-MO | 5′- TCAAATAAATGCTTACTTGTCGTCT-3′ | |  |
| Ze-nxhl-ATG-MO | 5′- GCGTGTAAGATTACGTCATTGCCTT-3′ | |  |
| Contro-MO  (Ze-ptprb) | 5’- CCTCTTACCTCACATGCAATTTATA-3’ | |  |
| Ze-ptprb-E4I4-MO | 5’-AGGCTTTATTTCTCAGCATCGTAAT-3’ | |  |
| Ze-ptprb-ATG-MO | **5’-TTCATTCAACTTGTACTAACCTGCT-3’** | |  |
| RT-PCR | | | |
| Hu-VEPTP | FOR:CAGAAAATCAGAAGGACGCA  REV:ATACACCCGAATGGAGGC | | 55 |
| Ze-nxhl | FOR:CTACGCACAAGACGACAAATAGC  REV:GGAGTACCACCGAATGCCAG | | 55 |
| RIP Probes |  | |  |
| Hu-VEPTP | FOR1:TAATACGACTCACTATAGGGTTCGGGTGTATCAGAC  REV1:TCGAGATTCATGGGACAG  FOR2:GTTCGGGTGTATCAGAC  REV2:TAATACGACTCACTATAGGGTCGAGATTCATGGGACAG | | 60 |
| Ze-ptprb | FOR1:TAATACGACTCACTATAGGGGTTCAGTGATGCTAACGGAG  REV1: TTGAGGCTTTGTCTTATGTC  FOR2:TAATACGACTCACTATAGGGTTGAGGCTTTGTCTTATGTC  REV2: GTTCAGTGATGCTAACGGAG | | 60 |
| Ze-nxhl ChIRP probes | | | |
| 1808142A-P1  1808142A-P2  1808142A-P3  1808142A-P4  1808142A-P5  1808142A-P6  1808142A-P7  1808142A-P8  1808142A-P9  1808142A-P10  1808142A-P11  1808142A-P12  1808142A-P13  1808142A-P14  1808142A-P15  1808142A-P16  1808142A-P17  1808142A-P18 | GTAAGATTACGTCATTGCCT  CGTTCTCAAAACGAGAAGGC  CAAGGCTATTTGTCGTCTTG  TTCTGGAGTGAATTCAGGCA  GCGGAAATTGGTGTCTTTTC  CCCGAAAAGCTGACTGACAT  GGAAATTTCATGTGCACGGG  TGTGCATCAATTGAGCCAAT  TACTACTGCTTGGAGTACCA  ATAAGACTGTCGTAGGACCC  TCTCCAATTAGGTAGTATCC  CAACCTGGAGTTGAAGCTTT  ACTTTACAGTCATTTCTCCT  ATGGTCCTCTGAAAACTGGG  ATAATGCTTCTCGTACGTCC  GATTCCACCAAGAATCCGAA  CGATGTGCAAATCCCTAACG  TCCTGTACACAGCGCTAAAA | |  |

Note: Ze represents zebrafish. Hu represents human. GP represents golden pompano.
