# Supplementary material for: Nxhl Controls Angiogenesis by Targeting VE-PTP Through Interaction With Nucleolin
Source: Front Cell Dev Biol. 2021 Oct 11;9:728821. doi: 10.3389/fcell.2021.728821 (PMC8558974; doi:10.3389/fcell.2021.728821)
Supplement: Supplementary Figure 1 — Expression profile of introns in Golden Pompano embryo development. (A) 19 embryo development stages were collected and transcriptome-sequenced. The FPKM of introns were used to draw the heatmap. A boundary lines between OSP-MGS and LGS-YAPS stages. (B) GO cluster of the differentially expressed genes. (C) KEGG cluster of the differentially expressed genes. Numbers of 1–57 below the heatmap represent embryo samples. Every three numbers represent one embryo stage. For example, number 1–3 represents stage OSP, number 55–57 represents stage YAPS. [file Data_Sheet_1.docx]

***Nxhl* Control****s Angiogenesis by Targeting VE-PTP through Interaction with Nucleolin**

**SUPPLEMENTARY FIGURES**

**Figure S1.** **Expression profile of introns in Golden Pompano embryo development.** **A,** 19 embryo development stages were collected and transcriptome-sequenced. The FPKM of introns were used to draw the heatmap. A boundary lines between OSP-MGS and LGS-YAPS stages. **B,** GO cluster of the differentially expressed genes. **C,** KEGG cluster of the differentially expressed genes. Numbers of 1-57 below the heatmap represent embryo samples. Every three numbers represent one embryo stage. For example, number 1-3 represents stage OSP, number 55-57 represents stage YAPS.

**Figure S2. Confirmation of *nxhl* gene knockdown. A,** Schematic map of *nxhl* gene and the MO design and micro-injection strategy. 4 ng morphants per fish embryo was injected. **B,** The gel electrophoresis of PCR products of *nxhl* gene in control and *nxhl-*MO groups. Compared with control MO, *nxhl* MO shows quite weak expression of *nxhl* gene. **C,** Expression profile of *nxhl* at 6hpf and 3dpf in zebrafish embryo. hpf, hours post fertilization; dpf, days post fertilization.

**Figure S3. Phenotypes of *nxhl* zebrafish morphants.** 4 ng morphants per fish embryo was injected. **A,** Gross morphology at 3-dpf. Compared with control MO, knock down *nxhl* present pericardial oedema (red arrow) and caudal fin defects (blue arrow). **B,** Representative bright field and fluorescent images of *Tg(fli1a:EGFP)^y1^* embryos at 52-hpf after injection of *nxhl* morphants.

**Figure S4.** Knockdown efficiency of the siRNAs. Trails for the choose of siRNAs before the formal experiments were performed. Three siRNAs targeting the *nxhl* gene were designed and tested for the knockdown efficiency. The siRNA-2 with the highest knockdown efficiency (about 70%) was chosen to perform the subsequent experiments.

**Figure S5. Gene expression profile post knockdown of *nxhl* comparing with control.** Scatter of the differential expressed genes after injection of *nxhl* morphants

**Figure S6.** KEGG enrichment of genes differentially expressed after knockdown of *nxhl* gene comparing with control.

**Figure S7. Phenotypes of *ptprb* zebrafish morphants.** 4 ng morphants per fish embryo was injected. **A,** Effectiveness of *ptprb* knockdown was confirmed by RT-PCR and Sanger sequencing. The zebrafish *ptprb* gene was targeted by specific morpholino antisense to prevent proper splicing of exon 4 (E4I4-MO). Primers 3F and 5R interrogate the presence of wild type (non-mutant) transcripts or those in which exon 4 has been skipped. Below the diagram is a schematic depiction of the exon 4-skipped transcript in the E4I4-MO–injected embryos (124bp) as compared with control embryos (388bp). RT-PCR of *ptprb* transcript from control and E4I4-MO morpholino- injected embryos 3 days after fertilization, demonstrating skipping of exon 4. Sanger sequencing of both the wild type band and the exon 4-skipped band validating the wild type sequence and the exon 4-skipped sequence. **B,** Gross morphology at 50 hpf. Compared with control MO, knock down *ptprb* present pericardial oedema (red arrow). **C,** Representative bright field and fluorescent images of *Tg(fli1a:EGFP)^y1^* embryos at 50 hpf after injection of *ptprb* morphants.

**C**

GCTGGCTAGCATGGCGCACTACGCTCGTTGTTTGCAGGTCTGCCTTCTCGTTTTGAGAACGTATATTAACATACAAAGTAATTTAATTCAACGACTTCACGCAGTTCAAAGACAATGTCACAGACAACGTCTACGCACAAGACGACAAATAGCCTTGGTGTTGATGAGAAGACGGACGGCAACTGTGTGGTCACATCCGCGAGAGCACCGCTGGTGGGACGTCATTGTGCCTGAATTCACTCCAGAAGAGTTCATCCAGAATTTCCGAGTCTCGCGGGAGTCTTTCGAGTACATATGCCGCCGCCTCAGACATATGCTTGAACGAAAAGACACCAATTTCCGCTTGAGCGTACCTGTAAAGAAACGAGTTGCCATTGCGCTCTGCAAGCTCGCCACTGGCAGCGAGTATCGATATGTCAGTCAGCTTTTCGGGGTTGGCGTGAGCACTGTGTTCAACTGTGTGCAGGACTTCTGCAGTGCCGTCATTAAAATTCTTGTGCCCGTGCACATGAAATTTCCAAGTCCCGAAAAGCTGAAGGAGATGGCAGACGTCTTCGAGAATTGCTGGAACGTACCACAGTGTATTGGCTCAATTGATGCACACCACATCCCAATAATAGCGCCTGAAAAAAACCCTCGTGGCTATCTTAACCGAAAAGGCTGGCATTCGGTGGTACTCCAAGCAGTAGTAGACGGGAACGGGCTCTTCTGGGATTTATGCGTTGGTTTTTCTGGAAACCTAAGTGATGCAAGGGTCCTACGACAGTCTTATTTATGGAGTTTGTTGAGTGAAAGAGACCTGCTGAACCACAACAAAGTAGACATCTCCGGTTGTGATGTTGGATACTACCTAATTGGAGATTCAGCTTATCCCTTGCAGAACTGGTTGATGAAGCCGTTTCCCGATATTGGTGGACTGACCCCGCAGCAGGAAAGCTTCAACTCCAGGTTGAGTAGCGCCAGATCGGTTTCGGATCTGAGTTTCAAGAAGCTAAAGGCACGATGGCAGTGTCTCTTCAGGAGAAATGACTGTAAAGTGGAGCTGGTGAAGAAGATGGCTTTGACCTGCTGCGTGCTACACAATATCTGTGAGGAAAAAGGAACCCAGTTTTCAGAGGACCATTCAACTGACCATCTGAACTTGCAGCCCCCTGTTCAGGAATTTATAGAGAATGGACAACCAGAAGGGACGGACGTACGAGAAGCATTATCAGACTTCTTTATCAGACAGAGTGATATTATTCAAGTAGTAGTTTAAGGTACCGAG

**Figure S8.** **Identification of pcDNA3.1-*nxhl* expression plasmid. A,** PCR amplification of *nxhl* gene（1267 bp, including enzyme cutting sites NheI and KpnI, and protective bases）. **B,** Four monoclones were selected by PCR and used for sequencing identification. **C,** Sequencing result of *nxhl* gene. The green underline is the fragment inserted into the vector, the vector sequence is on both sides. The red underline is the sequencing result of enzyme cutting site, which shows that the target sequence has been correctly constructed into the vector.

ATGGCGCACTACGCTCGTTGTTTGCAGGTCTGCCTTCTCGTTTTGAGAACGTATATTAACATACAAAGTAATTTAATTCAACGACTTCACGCAGTTCAAAGACAATGTCACAGACAACGTCTACGCACAAGACGACAAATAGCCTTGGTGTTGATGAGAAGACGGACGGCAACTGTGTGGTCACATCCGCGAGAGCACCGCTGGTGGGACGTCATTGTGCCTGAATTCACTCCAGAAGAGTTCATCCAGAATTTCCGAGTCTCGCGGGAGTCTTTCGAGTACATATGCCGCCGCCTCAGACATATGCTTGAACGAAAAGACACCAATTTCCGCTTGAGCGTACCTGTAAAGAAACGAGTTGCCATTGCGCTCTGCAAGCTCGCCACTGGCAGCGAGTATCGATATGTCAGTCAGCTTTTCGGGGTTGGCGTGAGCACTGTGTTCAACTGTGTGCAGGACTTCTGCAGTGCCGTCATTAAAATTCTTGTGCCCGTGCACATGAAATTTCCAAGTCCCGAAAAGCTGAAGGAGATGGCAGACGTCTTCGAGAATTGCTGGAACGTACCACAGTGTATTGGCTCAATTGATGCACACCACATCCCAATAATAGCGCCTGAAAAAAACCCTCGTGGCTATCTTAACCGAAAAGGCTGGCATTCGGTGGTACTCCAAGCAGTAGTAGACGGGAACGGGCTCTTCTGGGATTTATGCGTTGGTTTTTCTGGAAACCTAAGTGATGCAAGGGTCCTACGACAGTCTTATTTATGGAGTTTGTTGAGTGAAAGAGACCTGCTGAACCACAACAAAGTAGACATCTCCGGTTGTGATGTTGGATACTACCTAATTGGAGATTCAGCTTATCCCTTGCAGAACTGGTTGATGAAGCCGTTTCCCGATATTGGTGGACTGACCCCGCAGCAGGAAAGCTTCAACTCCAGGTTGAGTAGCGCCAGATCGGTTTCGGATCTGAGTTTCAAGAAGCTAAAGGCACGATGGCAGTGTCTCTTCAGGAGAAATGACTGTAAAGTGGAGCTGGTGAAGAAGATGGCTTTGACCTGCTGCGTGCTACACAATATCTGTGAGGAAAAAGGAACCCAGTTTTCAGAGGACCATTCAACTGACCATCTGAACTTGCAGCCCCCTGTTCAGGAATTTATAGAGAATGGACAACCAGAAGGGACGGACGTACGAGAAGCATTATCAGACTTCTTTATCAGACAGAGTGATATTATTCAAGTAGTAGTTGGGGGTGGAGGCTCTGATTACAAGGATGACGACGATAAGTAA

**Figure S9.** **Identification of pcDNA3.1-*nxhl*-Flag expression plasmid. A,** PCR amplification of *nxhl*-Flag gene（1306 bp, including enzyme cutting sites NheI and KpnI, and protective bases）based on pcDNA3.1-nxhl constructs. **B,** Sequencing result of *nxhl*-Flag fragment. The green underline is the fragment inserted into the vector, which shows that the target sequence has been correctly constructed into the vector.

**Figure S10. SDS-PAGE** [**electrophoresis**](app:ds:electrophoresis) **of *Ptprb* and *Nxhl* protein.** *Ptprb* and *Nxhl* protein was expressed in pet-b2m Rosetta expression system with [kanamycin](app:ds:kanamycin) [resistance](app:ds:resistance), respectively. Ptprb (33 kDa) and *Nxhl* (62 kDa) was successfully expressed and the purity of the recombinant protein was 85%. The purified protein was used to immunize rabbits to produce antibody, respectively. M, protein marker. 1, whole bacterial protein. 2, pet-b2m-*Nxhl* protein induced by IPTG. 3 and 4 pet-b2m-ptprb protein induced by IPTG.

**Figure S11.** **Silencing of Harbi1 (*nxhl*) not only decreases the angiogenesis of cancer cell lines but inhibits migration and EMT of HCC cells. A,** Silencing of Harbi1 (*nxhl*)gene inhibits angiogenesis of two cancer cell lines in vitro. HUVEC cells were plated on matrigel-coated plates at a density of 1.5 × 10^5^ cells/well and incubated for 24 h before the cell culture supernatant of si-Harbi1 (*nxhl*) treated non-small-cell lung carcinoma (A549) and colon carcinoma (HCT116) cell lines was added, respectively. Representative images of tube formation in si-Harbi1 (*nxhl*) treated A549 cells. Representative images of tube formation in si-Harbi1 (*nxhl*) treated HCT116 cells. The data represent as mean±SEM from three independent experiments. Scale bars, 100 μm. Images were taken 24h after addition of the supernatant. Tube networks were quantified using the ImageJ software and expressed as branches. **B,** Silencing of Harbi1 (*nxhl*) inhibits migration and EMT of HCC cells. Representative images of migrated cells stained with crystal violet and inhibition of migration in si-Harbi1 (*nxhl*) treated SMMC7721 cell (left) and HUVECs (right). Representative images of colony formation cells stained with crystal violet and inhibition of colony forming in si-Harbi1 (*nxhl*) treated SMMC7721 cell. **C,** Silencing of Harbi1 (*nxhl*) inhibits SMMC7721 cell growth. MTT method was used to test the cell proliferation. **D,** Harbi1 gene expression in HCC samples based on the TCGA data. The data represent as mean±SEM from three independent experiments. **p*<0.05, ***p*< 0.001, ****p*< 0.0001 represents statistically significant.

D
